# Aspirin modulates generation of procoagulant phospholipids in cardiovascular disease, by regulating LPCAT3

**DOI:** 10.1016/j.jlr.2024.100727

**Published:** 2024-12-12

**Authors:** Majd B. Protty, Victoria J. Tyrrell, Ali A. Hajeyah, Bethan Morgan, Daniela Costa, Yong Li, Anirban Choudhury, Rito Mitra, David Bosanquet, Alex Reed, Iuliia K. Denisenko, Katsuyuki Nagata, Hideo Shindou, Benjamin F. Cravatt, Alastair W. Poole, Takao Shimizu, Zaheer Yousef, Peter W. Collins, Valerie B. O’Donnell

**Affiliations:** 1 Systems Immunity Research Institute, Cardiff University, Cardiff, UK; 2 Bristol Platelet Group, School of Physiology, Pharmacology & Neuroscience, University of Bristol, Bristol, UK; 3 Morriston Cardiac Centre, Swansea Bay University Health Board, Swansea, UK; 4 Department of Cardiology, University Hospital of Wales, Cardiff, UK; 5 Department of Vascular Surgery, Aneurin Bevan University Health Board, Cwmbran, UK; 6 Department of Chemistry, The Scripps Research Institute, San Diego, CA; 7 National Center for Global Health and Medicine, Tokyo, Japan

**Keywords:** lipidomics, thrombosis, phospholipids, acute coronary syndrome, platelets

## Abstract

Enzymatically oxygenated phospholipids (eoxPL) from lipoxygenases (LOX) or cyclooxygenase (COX) are prothrombotic. Their generation in arterial disease, and their modulation by cardiovascular therapies is unknown. Furthermore, the Lands cycle acyl-transferases that catalyze their formation are unidentified. eoxPL were measured in platelets and leukocytes from an atherosclerotic cardiovascular disease (ASCVD) cohort and retrieved human arterial thrombi from three anatomical sites. The impact of age, gender, and aspirin was characterized in platelets from healthy subjects administered low-dose aspirin. The role of lysophosphatidylcholine acyltransferase 3 (LPCAT3) in eoxPL biosynthesis was tested using an inhibitor and a cell-free assay. Platelets from ASCVD patients generated lower levels of COX-derived eoxPL but elevated 12-LOX-diacyl forms, than platelets from healthy controls. This associated with aspirin and was recapitulated in healthy subjects by aspirin supplementation. P2Y12 inhibition had no impact on eoxPL. LPCAT3 inhibition selectively prevented 12-LOX-derived diacyl-eoxPL generation. LPCAT3 activity was not directly altered by aspirin. P2Y12 inhibition or aspirin had little impact on eoxPL in leukocytes. Complex aspirin-dependent gender and seasonal effects on platelet eoxPL generation were seen in healthy subjects. Limb or coronary (ST-elevation myocardial infarction, STEMI) thrombi displayed a platelet eoxPL signature while carotid thrombi had a white cell profile. EoxPL are altered in ASCVD by a commonly used cardiovascular therapy, and LPCAT3 was identified as the acyltransferase generating aspirin-sensitive 12-LOX diacyl forms. These changes to the phospholipid composition of blood cells in humans at risk of thrombosis may be clinically significant where the procoagulant membrane plays a central role in driving elevated thrombotic risk.

## Graphical abstract

**Figure:**
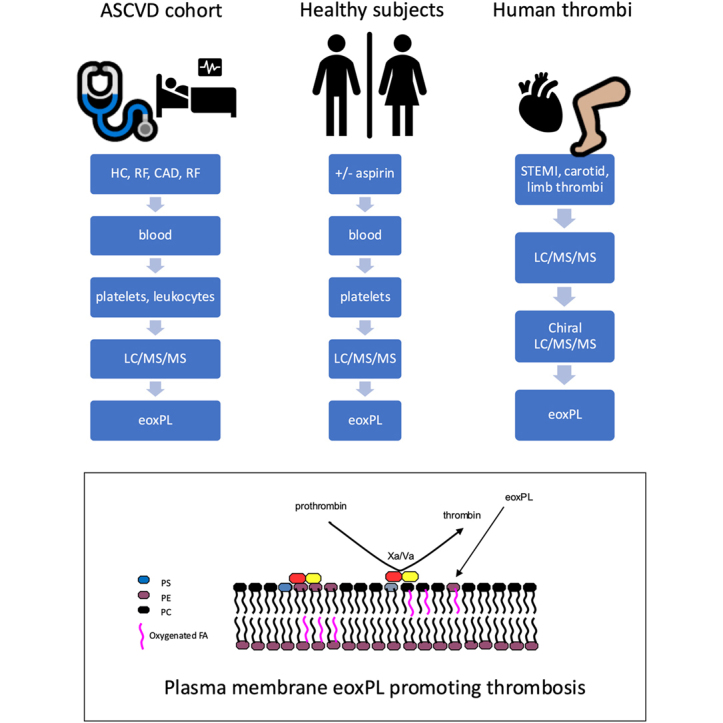

Coagulation factor activity, to form an occlusive thrombus, requires the presence of an electronegative procoagulant membrane, comprising phosphatidylserine (PS) and phosphatidylethanolamine (PE). In this model, resting cells maintain PS and PE on the inner plasma membrane leaflet, but on activation (eg thrombin activation of platelets) they externalize up to 7%–10% via scramblase (1, 2). Through associating with calcium and supported by PE, the PS headgroup supports binding and activity of coagulation factors, ultimately enabling formation of thrombin from prothrombin (3, 4, 5, 6). In recent years, a role for “enzymatically oxidized phospholipids” (eoxPL), in particular hydroxyeicosatetraenoic acids (HETEs) attached to PE or phosphatidylcholine (PC) in coagulation has been shown (7, 8). HETE-PL are acutely generated in ng amounts on platelet or leukocyte activation and a proportion become externalized at the outer membrane (5, 9, 10, 11, 12). Mechanistic studies demonstrated that eoxPL act to support PS on the membrane surface through mediating biophysical changes (13). Calcium binding is increased and accessibility of the PS headgroup to interact with factors appears to be enhanced, supporting increased thrombin generation (8, 13). The role of eoxPL in driving coagulation in vivo has only been examined in a small number of studies so far. These include venous thrombosis in mouse models and antiphospholipid syndrome in humans (8, 14). Mice lacking the lipoxygenase (LOX) enzymes that generate eoxPL (either *Alox15* or *Alox12*) generate smaller venous clots and bleed longer in challenge models (8, 14). Recently, a role for the LOX enzymes that generate eoxPL in promoting abdominal aortic aneurysm (AAA) in mice was demonstrated, and eoxPL were detected in human and murine AAA lesions (15).

Up to now, the generation and action of eoxPL in atherosclerotic cardiovascular disease (ASCVD), particularly in human coronary disease, has not been studied, and their role in regulating coagulation in this disease is unknown. Acute coronary syndrome (ACS) is a common manifestation of ASCVD. It leads to ischemic heart disease and is associated with high rates of mortality, recurrent infarction, and other complications (16, 17, 18, 19; https://heartuk.org.uk/press/press-kit/key-facts-figures). Inflammation is a central feature of ACS, where plaque rupture occurs along with recruitment of platelets (20, 21), leukocytes (22) and tissue factor upregulation (23, 24, 25, 26, 27). During thrombo-inflammation, coagulation leads to an occlusive arterial thrombus (28). Despite the use of antiplatelet therapy, rates of stroke, myocardial infarction, and cardiovascular death remain relatively high during the first year following diagnosis (29, 30). This suggests that there are other factors involved beyond platelets (29, 30, 31). A potential player is the coagulation cascade which causes formation of thrombin (32), driven by procoagulant membranes containing eoxPL.

While it is known that Lands cycle enzymes play a role in generation of eoxPL, to date, the specific acyl-CoA synthetases (ACSLs) and lysophospholipid acyl transferases (LPLATs) involved are so far uncharacterized (33). Delineating which enzymes regulate eoxPL generation in human blood cells could provide new targets for modulating eoxPL in vivo.

To test the involvement of eoxPL in arterial disease, we determined their profile in the membranes of platelets and leukocytes in ACS, and following aspirin supplementation in healthy subjects. A role for lysophosphatidylcholine acyltransferase 3 (LPCAT3); also known as LPLAT12 (34)) in regulating generation of LOX-derived eoxPL was tested. In a healthy cohort, we determined the impact of aspirin, gender, seasonality, and cell activation on eoxPL generation. Finally, the molecular species of eoxPL in human arterial thrombi were characterized.

## Materials and methods

### Participants

#### Healthy cohort

Healthy volunteers (n = 28) were recruited from the workplace (14 males, 14 females; age range 20–50 years). Ethical approval, which included informed consent, was from Cardiff University, Schools of Medicine and Dentistry Research Ethics Committee (SMREC16/02), and the study is registered as NCT05604118 (ClinicalTrials.org). Following a 2-week period free from nonsteroidal antiinflammatory drugs, peripheral blood was obtained, and platelets isolated as outlined below. Participants were commenced on aspirin 75 mg once daily for seven days and then provided a repeat sample. Two (one male and one female) participants were unable to provide a sample post-aspirin. Following a 2-month period, a subset (7 males and 7 females) returned and provided repeat samples pre-aspirin and post-aspirin, and again a third time 2 months later.

#### Clinical cohort

Participants were recruited from Cardiff University and Cardiff and Vale University Health Boards. Ethical approval was from the Health and Care Research Wales (HCRW, IRAS 243701; REC reference 18/YH/0502). Study groups of at least 20 were aimed for the study based on a previous study in venous thrombosis (8). Age and gender-matched individuals were recruited as follows: (i) ACS: participants were identified on in-patient cardiology wards using diagnostic tests (ischemic electrocardiogram changes, raised troponin level above normal laboratory defined range) and clinical assessment by the cardiology team. All were recruited within 48 h of the index event prior to any revascularization/angioplasty. (ii) Significant coronary artery disease (CAD): Patients attending for an elective coronary angiogram to assess for the symptoms of stable angina in the absence of a history of ACS were recruited. Coronary angiography demonstrated lesions requiring revascularization on anatomical/physiological criteria as defined by guidelines from the European Society for Cardiology (ESC, 2018) (35). (iii) Risk-factor controls with no significant CAD (RF): This group includes patients attending for a diagnostic coronary angiogram with any risk factors for ischemic heart disease (a clinical diagnosis of hypertension requiring therapy, diabetes types 1 or 2, hypercholesterolemia [total cholesterol > 6 mmol/L], smoking, chronic kidney disease stage 3 or more, or combination thereof) but whose coronary angiogram demonstrates no significant coronary artery disease, defined as not requiring revascularization on anatomical/physiological criteria as per the European Society for Cardiology 2018 guidelines (35). (iv) Healthy controls (HC): Participants had no history of ischemic heart disease or its risk factors, were never-smokers, and were not on antiplatelet agents, anticoagulants, antihypertensives, or statins. They were identified from the workplace or were volunteers from partner studies such as “HealthWise Wales” (36). Clinical characteristics are in supplemental Table S1. Inclusion criteria were aged >18 years, acute coronary syndrome in ACS group, and no history of ACS in the others. Exclusion criteria were the following: diagnosis of infective endocarditis or atrial fibrillation, or inability to consent to study. Here, 90 patients were recruited: HC, n = 24, RF, n = 23, CAD, n = 19 and ACS, n = 24. Blood samples were collected by peripheral venepuncture as outlined below, by one individual and all samples were transferred to the laboratory within 10 min. All human studies abided by the Declaration of Helsinki principles.

### Platelet isolation

Whole blood was taken from the antecubital vein using a 21G butterfly into a 50 ml syringe containing acidified citrate dextrose (ACD; 85 mM trisodium citrate, 65 mM citric acid, 100 mM glucose, pH 5.0) at a ratio of 8.1 parts whole blood to 1.9 parts ACD, as described previously (8), and centrifuged at 250 *g* for 10 min at 20°C. The platelet-rich plasma was collected and centrifuged at 1,000 *g* for 8 min at 20°C. Platelet poor plasma was removed. The platelet pellet was resuspended in Tyrode's buffer (134 mM NaCl, 12 mM NaHCO_3_, 2.9 mM KCl, 0.34 mM Na_2_HPO_4_, 1.0 mM MgCl_2_, 10 mM Hepes, 5 mM glucose, pH 7.4) containing ACD (9:1, v/v). The platelets were washed by centrifuging at 1,000 *g* for 8 min at 20°C then resuspended in Tyrode's buffer at a concentration of 2 × 10^8^ ml^−1^. Platelets were activated at 37°C in the presence of 1 mM CaCl_2_, 0.2 unit·ml^−1^ thrombin (Sigma-Aldrich) for 30 min with occasional inversion. In some experiments, 10 μM (*R*)-HTS-3 was preincubated with washed platelets prior to activation (37).

Platelets were isolated in an air-conditioned room maintained at a constant temperature of 22°C. They were isolated, activated, and then extracted on the same day, taking care that the same internal standard (IS) batches were used for all samples in each batch. It was essential to process them to extracts on the same day of platelet isolation so that enzymatic reactions were stopped prior to storage. Once they were all prepared, they were stored at −80°C and then analyzed using LC/MS/MS as one large batch. Harmonization material was not used.

### Leukocyte isolation

Leukocytes were isolated from 20 ml citrate-anticoagulated whole blood as described (8). Briefly, 20 ml of blood was mixed with 4 ml of 2% citrate and 4 ml of HetaSep (STEMCELL Technologies) and allowed to sediment for 45 min. The upper plasma layer was recovered and centrifuged at 250 *g* for 10 min at 4°C. The pellet was resuspended in ice-cold 0.4% trisodium citrate/PBS and centrifuged at 250 *g* for 5 min at 4°C. Erythrocytes were removed by hypotonic lysis (0.2% hypotonic saline). Leukocytes were resuspended in Krebs buffer (100 mM NaCl, 48 mM Hepes, 5 mM KCl, 1 mM sodium dihydrogen orthophosphate dihydrate, and 2 mM glucose) at 4 × 10^6^/ml. For activation, 4 × 10^6^ leukocytes were incubated at 37°C with 10 μM A23187 and 1 mM CaCl_2_, for 30 min with occasional inversion, prior to lipid extraction.

### Human thrombi

The study took place between Cardiff University, Cardiff and Vale University Health Board (CVUHB) and Aneurin Bevan University Health Board (ABUHB). Ethical approval was from Health and Care Research Wales (HCRW, IRAS 243701; REC reference 18/YH/0502). Patients were identified by the collaborating vascular/cardiology teams from their operating patient lists. There were no changes to the standard operating procedure and no additional surgical/interventional steps were carried out. Some procedures were time-critical, and ethical approvals allowed the research team to seek retrospective consent in the 48 h after the procedure. This was in the form of written informed consent which included permission to record participants’ medical history from hospital notes. If consent was declined, stored samples were discarded. Inclusion criteria were >18 years and undergoing vascular surgery or percutaneous transluminal intervention to treat arterial thrombotic disorders, where there was a clinical indication to remove diseased tissue. Exclusion criteria were inability to consent within specified time frame. Thrombi were obtained from patients as follows: (i) Coronary thrombi were collected from patients presenting with ST-elevation myocardial infarction (STEMI) to the Cardiology Department in Cardiff and Vale University Health Board who underwent emergent percutaneous coronary intervention. Patients were diagnosed on arrival based on electrocardiographic features of ST-segment elevation in more than two leads corresponding to a myocardial muscle territory. Following this, patients were immediately prepared for coronary angioplasty. A thrombus aspiration catheter (Export Advance™, Medtronic, Ireland) was advanced through the peripheral vascular access (radial artery) to the coronary artery and the thrombus retrieved. This was washed with saline using a cell sieve, transferred to a sterile cryovial and immediately snap-frozen on dry ice prior to transfer to −80°C. (ii) carotid thrombi were collected from patients at the Vascular Department at Aneurin Bevan University Health Board identified from routine operating lists, who were undergoing carotid endarterectomy, typically following an ischemic stroke/transient ischemic attack with evidence of >50% stenosis of the appropriate internal carotid artery on duplex ultrasonography. (ii) Limb thrombi were taken from patients with acute limb ischemia requiring either embolectomy, thrombectomy, arterial bypass, or amputation. For carotid and limb clots, the clot was retrieved using standard open surgical techniques, placed into cryovials and immediately snap-frozen on dry ice prior to transfer to −80°C freezer for storage. supplemental Tables S2 and S3 show clinical demographics of patients including biochemical and hematological characteristics.

### Expression of LPLAT12 (LPCAT3) in CHO-K1 cells

After 48 h of transfection with each empty and FLAG-human LPCAT3 (LPLAT12) complementary DNA in pCXN2 by using Lipofectamine 2000 (Thermo Fisher Scientific, Waltham, MA), cells from 10-cm dishes were scraped into 1 ml of ice-cold buffer containing 100 mM Tris-HCl (pH 7.4), 300 mM sucrose, and a proteinase inhibitor mixture, Complete (Roche Diagnostics, Indianapolis, IN), and then sonicated three times on ice for 30 s. After centrifugation for 10 min at 800 *g*, each supernatant was collected and centrifuged at 100,000 *g* for 1 h. The resulting pellets were resuspended in buffer containing 20 mM Tris-HCl (pH 7.4). Protein concentration was measured by the method of Bradford (PMID: 942051), using a commercially prepared protein assay solution (Bio-Rad) and BSA, fraction V, fatty acid-free; Sigma-Aldrich) as a standard.

### Western blot analysis

The microsomal fraction (5 μg each of 100,000 *g* pellets) was resolved by 10% SDS-PAGE and transferred to a Hybond enhanced chemiluminescence nitrocellulose membrane (GE HealthCare UK Ltd.). The membrane was stained with Ponceau S (Sigma-Aldrich) to visualize the total protein levels. After destaining, the membrane was blocked with 5% skim milk, incubated with anti-FLAG M2-Peroxidase mAb (Sigma-Aldrich). After washing, the membrane was exposed to enhanced chemiluminescence reagents (GE HealthCare UK Ltd.) and the proteins were detected using ImageQuant LAS 500 (GE HealthCare).

### LPCAT assay

Proteins (0.1 μg) were incubated with 100 mM Tris-HCl (pH7.4), 20 μM C16:0 d31LPC, 20 μM C20:4-CoA, and 0.015% Tween-20 with or without 1 mM aspirin for 10 min at 37°C. The reaction was stopped by the addition of 300 μl of CHCl_3_:MeOH = 1:2 (v/v). Internal standard (50 μl of 0.2 μM PC 14:0/14:0) was added, and total lipids were extracted by Bligh-Dyer method (38). Lipids were analyzed by using LCMS-8060 (Shimadzu, Kyoto, Japan). All lipid reagents were purchased from Avanti Polar Lipids, Inc. (Birmingham, AL).

### Lipid extraction and LC/MS/MS

(i) Platelets and leukocytes. IS were added (10 ng 1,2-dimyristoyl-PE and -PC, Avanti Polar Lipids) to leukocytes or platelets, then lipids extracted using a solvent mixture [1 M acetic acid, 2-propanol, hexane (2:20:30)] at a ratio of 2.5 ml of solvent to 1 ml sample, vortexing for 1 min, then adding 2.5 ml of hexane. Following 1 min vortexing and centrifugation (400 *g*, 5 min), lipids were recovered in the upper hexane layer. The samples were then reextracted by the addition of hexane (2.5 ml/ml sample) followed by further vortexing and centrifugation, as above. The combined hexane layers were dried under vacuum and stored at −80°C until analysis using LC/MS/MS.
(ii) Clot lipid extraction. Clots were retrieved onto dry ice and divided with a scalpel if > 200 mg by visual estimate. Clots were weighed, transferred into 1.5 ml tubes, and placed on wet ice. Since these contain high levels of prooxidant hemoglobin, ice cold antioxidant buffer (0.5 ml PBS containing 100 μM diethylenetriamine pentaacetic acid, 100 μM butylated hydroxytoluene, and 7.5 μM acetaminophen) was added to minimize artefactual oxidation that could take place during tissue processing, along with IS (10 ng DMPC/1,2-dimyristoyl-sn-glycero-3-phosphatidylcholine, 1,2-dimyristoyl-sn-glycero-3-phosphatidylethanolamine) and 8–10 ceramic beads (1.4 mm). To reduce unstable lipid hydroperoxides, SnCl_2_ (100 mM) was added. The tubes were placed in a Bead Ruptor Elite™ (Omni) and tissue homogenization carried out at 4 m/s for 20 s at 4°C. Homogenized clot samples (0.5 ml) were transferred to glass tubes containing 2.5 ml ice-cold methanol (MeOH) and placed on ice. A further 0.5 ml antioxidant buffer was added. A modified Bligh and Dyer lipid extraction method was carried out by adding 1.25 ml chloroform per sample. The mixture was vortexed for 1 min, and samples were placed on ice for 30 min. Following this, 1.25 ml of chloroform was added and the mixture vortexed for 1 min. Briefly, 1.25 ml of water was added, solvent mixture vortexed for 1 min, and then centrifuged at 400 *g* for 5 min at 4°C to support phase separation. The bottom layer was collected into fresh vials using glass Pasteur pipettes and samples were evaporated to dryness using a Rapidvap vacuum evaporation system (Labconco). Lipids were resuspended in 100 μl MeOH, transferred to HPLC vials, and stored at −80°C until analysis using LC/MS/MS.

For LC/MS/MS, samples were separated on a C18 Luna, 3 μm, 150 mm × 2 mm column (Phenomenex) gradient of 50%–100% solvent B for 10 min followed by 30 min at 100% B (Solvent A: MeOH:acetonitrile:water, 1 mM ammonium acetate, 60:20:20; Solvent B: MeOH, 1 mM ammonium acetate) with a flow rate of 200 μl/min. Products were analyzed in multiple reaction monitoring (MRM) mode, on a Q-Trap 6,500 or 7,500 (Sciex) operating in negative mode. The peak area for analytes was integrated and normalized to the IS area. For quantification of HETE-containing PL, standard curves were generated using PC and PE 18:0a/HETE, as described previously (39). Limit of quantitation (LOQ) is defined as signal:noise of 5:1 with at least 6 data points across a peak. Settings for 6,500 were dwell time of 75 msec, declustering potential −50 V, entrance potential−10 V, collision energy−38 V, and collision cell exit potential −11 V. Settings for 7,500 were spray Voltage −3,500, collision energy-44, entrance potential-10, and collision cell exit potential-19. MRM transitions for 48 platelet eoxPL were analyzed for platelets from the healthy cohort (40), while for the clinical samples and clots, where both platelets and white blood cells were analyzed, an MRM list that comprised all the known HETE-PL isomers was used. This is because these cells will also make eoxPL from 15- and 5-LOX, which are not present in platelets. For many of the platelet MRM list, due to the absence of full structural information and synthetic standards, names are annotated only in part as putative structures, by omitting the position of hydroxyl group or the specific oxygenated functional group, and they are presented as area of analyte:internal standard (A/IS, cps). The list of MRM transitions used are in supplemental Table S4 (healthy cohort) and 5 (clinical study).

### LC/MS/MS of 12-HETE and TXB2

Platelet lipid extracts from healthy subjects were analyzed for 12-HETE and TXB2 using LC/MS/MS. However, since samples had already been extracted for analysis, this was a post hoc estimate, meaning that IS (5 ng of each of 12S-HETE-*d8* and TXB2-*d4* (Cayman Chemical)) were added after lipid extraction. Thus, variability in extraction efficiency would not be accounted, and data are expressed as A/IS (area, cps). Also, insufficient volumes of all samples were available due to prior assays, and so only a subset of 14 samples were measured. Samples were processed on a C18 Spherisorb ODS2, 5 μm, 150 × 4.6-mm column (Waters, Hertfordshire, UK) using a gradient of 50%–90% B over 10 min (A, water:acetonitrile:acetic acid, 75:25:0.1; B, MeOH:acetonitrile:acetic acid, 60:40:0.1) with a flow rate of 1 ml/min. LC/MS/MS was by electrospray ionization on a Q-Trap 4,000 (Sciex, UK) operating in negative ion mode, monitoring for MRM transitions for 12-HETE, TXB2, and their respective deuterated IS. Instrument settings are in supplemental Table S6. LOQ is defined as signal:noise of 5:1 with at least 6 data points across a peak.

### LC/MS/MS of LPCAT assay products

LPCAT assay products and internal standard (PC 14:0/14:0) were measured using an LCMS8060 instrument (Shimadzu). A total of 5 μl of sample was injected. Samples were separated by an ACQUITY UPLC BEH C8 1.7 μm 2.1 × 30 mm Column (Waters Co., Milford, MA) at 47°C using a gradient of solvent A (5 mM ammonium bicarbonate, Fujifilm) and solvent B (acetonitrile, Fujifilm) and solvent C (isopropanol, Fujifilm) under 0.5 ml/min flow condition. The gradient [time (A/B/C (%))] was programed as follows: 0 min (60/20/20)–0.5 to 3.45 min (20/40/40)–3.5 to 4.85 min (6/47/47)–4.9 to 5 min (60/20/20, start condition) in the assay measurement. Assay samples were measured in positive mode using selected reaction monitoring transitions (PC 28:0 678.5 → 184; PC 36:4-d31 813.5 → 184).

### Chiral LC/MS/MS of clot HETEs

Lipid extracts underwent alkaline hydrolysis followed by chiral LC/MS/MS as described previously (10, 41). Subsequently, 80 μl of lipid extract was dried under N_2_, then resuspended in 1.5 ml 2-propanol and vortexed for 1 min. In addition, 1.5 ml of 1 M NaOH was added followed by vortexing for 10 s. Samples were placed in a 60°C water bath for 30 min, and 140 μl of 12 M HCl was added to acidify to pH 3.0. Next, 3 ng 12(S)-HETE-*d8* was added to each sample, followed by the addition of 3 ml hexane, vortexing for 1 min, and centrifuging at 250 *g* for 5 min at 4°C. Lipids were recovered in the upper hexane layer. The aqueous portion was reextracted by addition of a further 3 ml hexane, vortexed for 1 min, and centrifuged at 250 *g* for 5 min at 4°C. The hexane layers were combined, and dried using a RapidVap (Labconco Corporation). Lipids were resuspended in 60 μl MeOH and transferred to an LC/MS/MS vial. The *S* and *R* enantiomers for HETE positional isomers were separated on a Chiralpak AD-RH, 5 μm, 150 × 4.6-mm column (Daicel, France) using an isocratic solvent (MeOH:H_2_O:glacial acetic acid, 95:5:0.1 v/v) with a flow rate of 0.3 ml/min over 25 min. Products were quantified by LC/MS/MS on an Sciex Q-Trap 4,000 in negative ion mode using MRM transitions for 15-, 12-, 11-, 8-, and 5-HETE, as well as 12(S)-HETE-*d8*, with instrument settings in supplemental Table S7.

### Statistical analysis

LC/MS/MS data were exported from Analyst v1.6.3 (Sciex) and analyzed using MultiQuant v3.0.3 (Sciex). Statistics and bioinformatics used the coding environment R (Open source, version 3.5.3; www.r-project.org). For heatmaps, mean values (ng/ml or cell number) were log10 transformed and plotted as intensity values using the Pheatmap package with lipid hierarchical clustering. Each lipid was annotated, using a color code, according to its presumed enzymatic origin (42). For plots, whiskers indicate 1.5 times the interquartile range shown with the median and all data points. Significance was determined using Mann-Whitney-Wilcoxon and Kruskal-Wallis tests for analysis of differences between more than two groups (astatsa.com). For paired samples, paired *t* test was used, where data were determined using Kolmogorov-Smirnov test to be normally distributed (socscistatistics.com), or Wilcoxon Signed-Rank Test where it was not. Basal samples were not included in statistical comparisons of activated samples since eoxPL were rarely detected without activation. For the purposes of statistical comparison, zeros were replaced with 50% of the LOQ value as calculated by a standard curve on the same instrument and column settings. If more than 70% of values were below LOQ, the lipid was removed. Multiple comparison adjustment was used for volcano plots, using Bonferroni correction. Principal component analysis used the singular value decomposition method provided by the base R “prcomp” function. Example chromatograms for all lipids detected are provided in supplemental Fig. S1. Data reporting are in line with ARRIVE (43) and STROBE (44) guidelines. Statistical analysis of the clinical cohort was mainly performed on data combined from both biological sexes. The females in the clinical study will largely be postmenopausal since their mean age was above 60 years, and it is well-known that cardiovascular risk of postmenopausal women is increased, and more in line with that of males. Also, there is a large predominance of males in the ASCVD groups, as would be expected due to the demographics of this disease. Due to this, in some groups there were insufficient females for statistical analysis. For comparison of the impact of aspirin, we also looked at males alone, noting that all females were on aspirin and so it was not possible to analyze this group. In contrast the healthy cohort subjects were all between 20 and 50 years old and are expected not to be post menopausal. Here, separate comparisons between male and female subjects were possible both on and off aspirin supplementation.

## Results

### Platelets from all patient groups generate higher levels of 12-HETE-PL but lower levels of 11- and 15-HETE-PL, than those from healthy controls

**Fig. 1:**
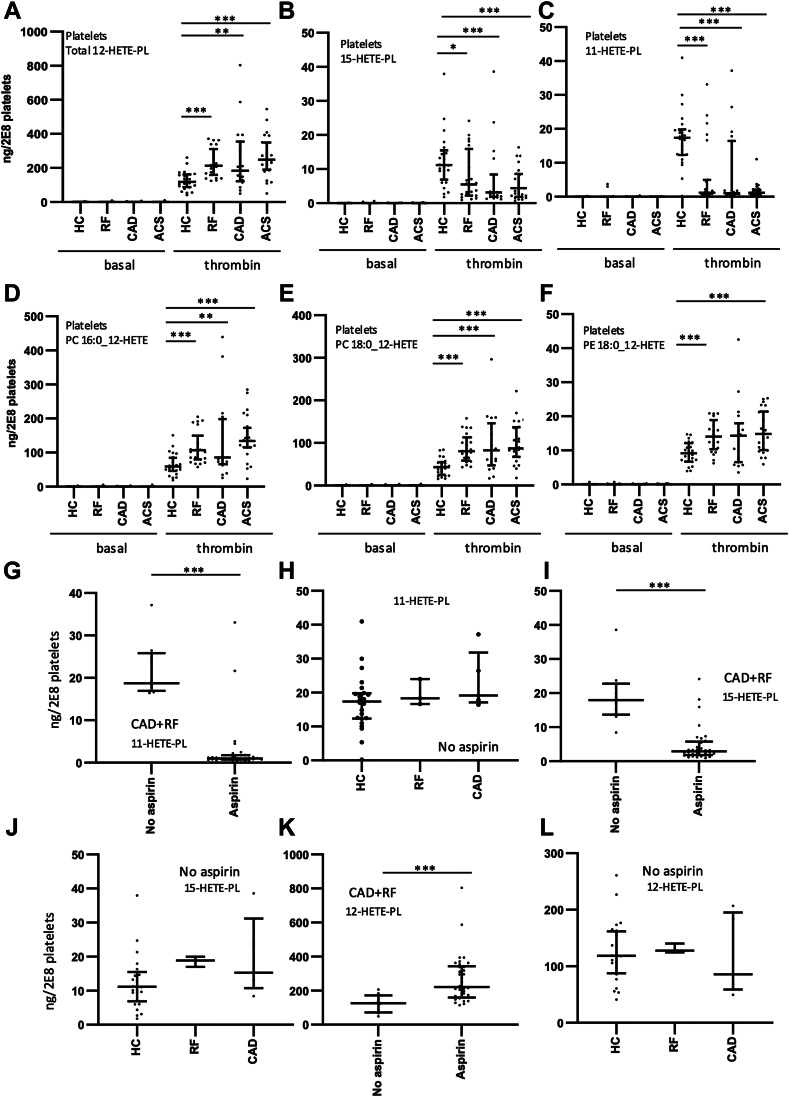
Aspirin reduces COX-1 generated eoxPL but increases 12-LOX generated acyl eoxPL in thrombin-activated platelets in ASCVD. Blood samples from a clinical cohort of ASCVD were collected, platelets (2 × 10^8^/ml) isolated, and then thrombin-activated (0.2 unit·ml^−1^ thrombin, 37°C, 30 min). Lipids were extracted and analyzed using LC/MS/MS and HETE-PL quantified. Individual isomers were quantified then totaled to give amounts as follows: Panel (A). 12-HETE-PL, Panel (B). 11-HETE-PL, and Panel (C). 15-HETE-PL. ACS: acute coronary syndrome (n = 24), CAD: coronary artery disease but no ACS (n = 19), RF: Risk factors with no significant coronary artery disease (n = 23), HC: Healthy control (n = 24). Panels (D–F). Lipids are displayed as individual diacyl-12-HETE-PL. Panel (G). 11-HETE-PL were compared in RF + CAD groups based on whether they were on aspirin or not (n = 34,8). Panel (H). 11-HETE-PL were compared in patients not on aspirin, in HC, RF, and CAD groups (n = 23, 3, 5, for HC, RF, and CAD, respectively). Panel (I). 15-HETE-PL were compared in RF + CAD groups based on whether they were on aspirin or not (n = 34, 8). Panel (J). 15-HETE-PL were compared in patients not on aspirin, in HC, RF, and CAD groups (n = 23, 3, 5, for HC, RF, and CAD, respectively). Panel (K). 12-HETE-PL were compared in RF + CAD groups based on whether they were on aspirin or not (n = 34, 8)). Panel (L). 12-HETE-PL were compared in patients not on aspirin, in HC, RF, and CAD groups (n = 23, 3, and 5, for HC, RF, and CAD, respectively). Statistical significance was tested with Mann-Whitney-Wilcoxon test for pairwise comparison after Kruskal-Wallis test for nonparametric analysis of variance on rank (∗*P* < 0.05, ∗∗*P* < 0.01, ∗∗∗*P* < 0.001). For Panels (H, K, and L), only CAD and HC groups were compared since only three patients were available in the RF group. eoxPL, enzymatically oxygenated phospholipid; COX, cyclooxygenase; ASCVD, atherosclerotic cardiovascular disease; ACS, acute coronary syndrome; CAD, coronary artery disease; LOX, lipoxygenase; HETE-PL, hydroxyeicosatetraenoic acid–phospholipid.

HETE-PL were quantified in platelets from patients with ACS, CAD, and RF basally and then following thrombin activation. The most abundant PE and PC species previously detected in platelets were measured using LC/MS/MS, specifically four PEs and two PCs with 18:0, 18:1, or 16:0, including both plasmalogen and acyl forms. Five HETE positional isoforms (5-, 8-, 11-, 12-, and 15-) of these were quantified (10). Little or no HETE-PL were detected basally; however, thrombin stimulated large increases, particularly the abundant 12-HETE-PL species formed via 12-LOX (10) (Fig. 1A). Other HETE-PL also were formed on activation, mainly 15- and 11-HETE-PL but also very low levels of 8-HETE-PL, while 5-HETE-PL were below limit of detection (Fig. 1B, C, supplemental Fig. S2A). Briefly, 12-HETE-PL were formed at significantly higher levels in activated platelets from all patient groups, than HC (Fig. 1A). The increase in 12-HETE-PL was due to higher levels of the three diacyl-12-HETE-PL, with no significant differences for plasmalogens (Fig. 1D–F, supplemental Fig. S2B–D). In contrast, 15- and 11-HETE-PL were significantly reduced in activated platelets from all groups compared to HC (Fig. 1B, C). Here, both plasmalogen and acyl-forms of HETE-PEs, and PCs were impacted equally (supplemental Figs. S2E–J, S3A–E). Subsequently, 8-HETE-PL in activated platelets were not different for patient groups versus HC (supplemental Fig. 2A). Overall, this indicates that diacyl-eoxPL from 12-LOX are significantly higher in patient groups, while both acyl and plasmalogen 15- and 11-HETE-PL species are reduced. The enzymatic origin of these lipids will be explored below.

### Aspirin supplementation associates with elevated 12- but reduced 11- and 15-HETE-PL in platelets from patient groups

In platelets, 15- and 11-HETE-PL likely are generated following cyclooxygenase 1 (COX-1) formation of 15- and 11-HETE, followed by their esterification into PL. To test this, the effect of aspirin supplementation was determined. All ACS, but no HC participants were taking aspirin (supplemental Table S8). In contrast, in both the RF and CAD groups, a small number were not on aspirin allowing direct comparison within these groups. Although patient numbers in some groups were relatively low, a significant suppressive effect of aspirin on both 11- and 15-HETE-PL generation was clearly observed, while for participants not on aspirin in the HC, RF, and CAD groups, there were no significant differences (Fig. 1G–J). Aspirin supplementation was also associated with a significant increase in 12-HETE-PL levels in the RF + CAD groups (Fig. 1K), while for participants not on aspirin, there was no impact of disease (Fig. 1L). Next, the individual isoforms of 12-HETE-PL in the RF + CAD groups were measured. In the case of 12-HETE-PL, two of the three diacyl isoforms were significantly increased in platelets by aspirin supplementation, while for all plasmalogens, there was no effect (supplemental Fig. S3F). Last, since all female RF and CAD patients were on aspirin, to test the effect of gender, males only were compared. As seen above, aspirin significantly increased platelet generation of diacyl 12-HETE-PL for RF + CAD males (supplemental Fig. S4). In contrast, reductions in 11- and 15-HETE-PL were seen for both acyl and plasmalogen forms in CAD and RF groups, with many PCs in the aspirin groups being below LOQ of our assay, and not quantifiable (supplemental Fig. S5). These data indicate that altered platelet eoxPL generation in ASCVD is driven by aspirin. This idea will be further tested in a healthy cohort, later. Using clinical data, eoxPL generation by platelets was also compared to either medication use (anticoagulants, P2Y12 inhibitors, and statins) or other diseases (diabetes, hypertension), but no correlations were noted.

### eoxPL generation was modulated by vascular disease in ionophore-activated leukocytes

**Fig. 2:**
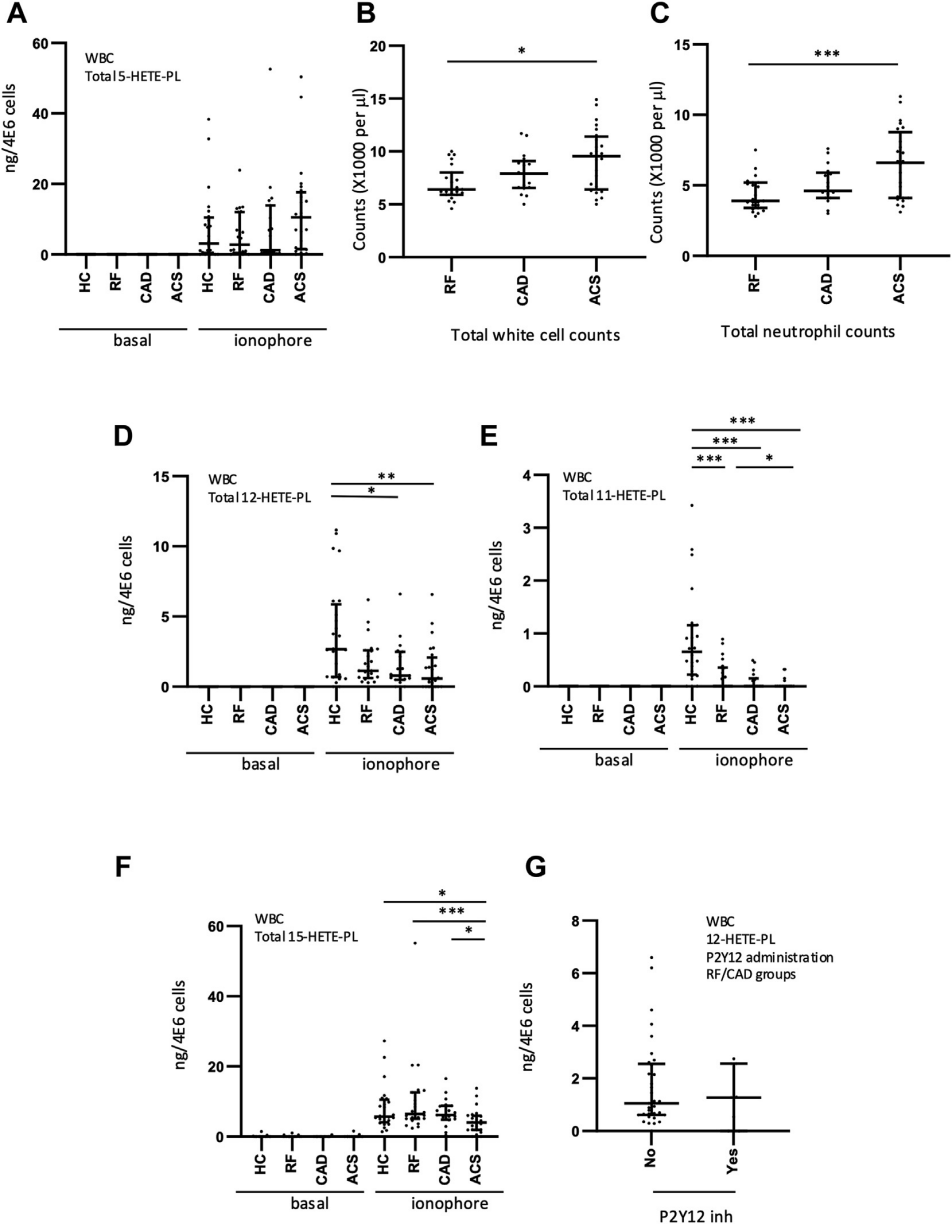
12-, 11-, and 15-HETE-PL in ionophore-activated leukocytes are lower in patients with ASCVD than healthy controls. Blood samples from a clinical cohort of ASCVD were collected, leukocytes (4 × 10^6^/ml) isolated, and then ionophore-activated (10 μM A23187, 37°C, 30 min). ACS: acute coronary syndrome (n = 24), CAD: coronary artery disease but no ACS (n = 19), RF: Risk factors with no significant coronary artery disease (n = 23), HC: Healthy control (n = 24). Lipids were extracted and analyzed using LC/MS/MS and HETE-PL quantified. Individual isomers were quantified then totaled to give amounts as follows: Panel (A): 5-HETE-PL. Panels (B, C). Leukocyte and neutrophil counts were obtained from clinical laboratory measurements, but were unavailable for the HC group. Panel (D): 12-HETE-PL, Panel (E): 11-HETE-PL, Panel (F): 15-HETE-PL. Panel (G): 12-HETE-PL levels in RF and CAD groups compared with/without P2Y12 inhibitor supplementation (n = 33 and 9, respectively). Statistical significance was tested with Mann-Whitney-Wilcoxon test for pairwise comparison after Kruskal-Wallis test for nonparametric analysis of variance on rank (∗*P* < 0.05, ∗∗*P* < 0.01, ∗∗∗*P* < 0.001). ASCVD, atherosclerotic cardiovascular disease; CAD, coronary artery disease; HETE-PL, hydroxyeicosatetraenoic acid–phospholipid.

Next, HETE-PL levels were analyzed in leukocyte isolates obtained from patients and healthy control subjects both basally and after ionophore activation, to determine the capacity of the cells to generate them. Generation of eoxPL by ionophore-activated leukocytes was demonstrated, particularly in healthy control samples (Fig. 2A). However, levels of eoxPL were lower than in platelets, with a higher degree of samples falling below assay LOQ for individual molecular species. Several HETE-PL formed on activation, mainly 15-, 12-, or 5-HETEs, with 11-HETE-PL being generated at very low levels (Fig. 2A, D–F, supplemental Figs. S6 and S7). Overall, total 5-HETE-PL (when normalized to leukocyte numbers) generated by 5-LOX showed a small nonsignificant trend to elevate in all groups (Fig. 2A, supplemental Fig. 6A). When cell counts for the patient groups were compared, a significant increase was seen for ACS versus RF for both total white cells and neutrophils (Fig. 2B, C). Thus in vivo, the amounts of 5-HETE-PE generated overall may be further elevated due to the higher cell counts. Generation of several 15, 12, or 11-HETE-PL species were significantly decreased in patient groups, versus HC (Fig. 2D–F, supplemental Figs. S6B, S7). We next examined if this finding was associated with drug treatments or coexisting conditions. While there were no significant differences seen for P2Y12 inhibitors (although not many patients were on these drugs), aspirin was associated with a small increase in 15-HETE-PL but no changes for 11-, 12-, or 5-HETE-PEs (Fig. G, supplemental Fig. S8A). Last, there were no associations with statins, hypertension or diabetes seen.

### Characterizing the impact of aspirin and gender on eoxPL generation by human platelets from healthy volunteers

**Fig. 3:**
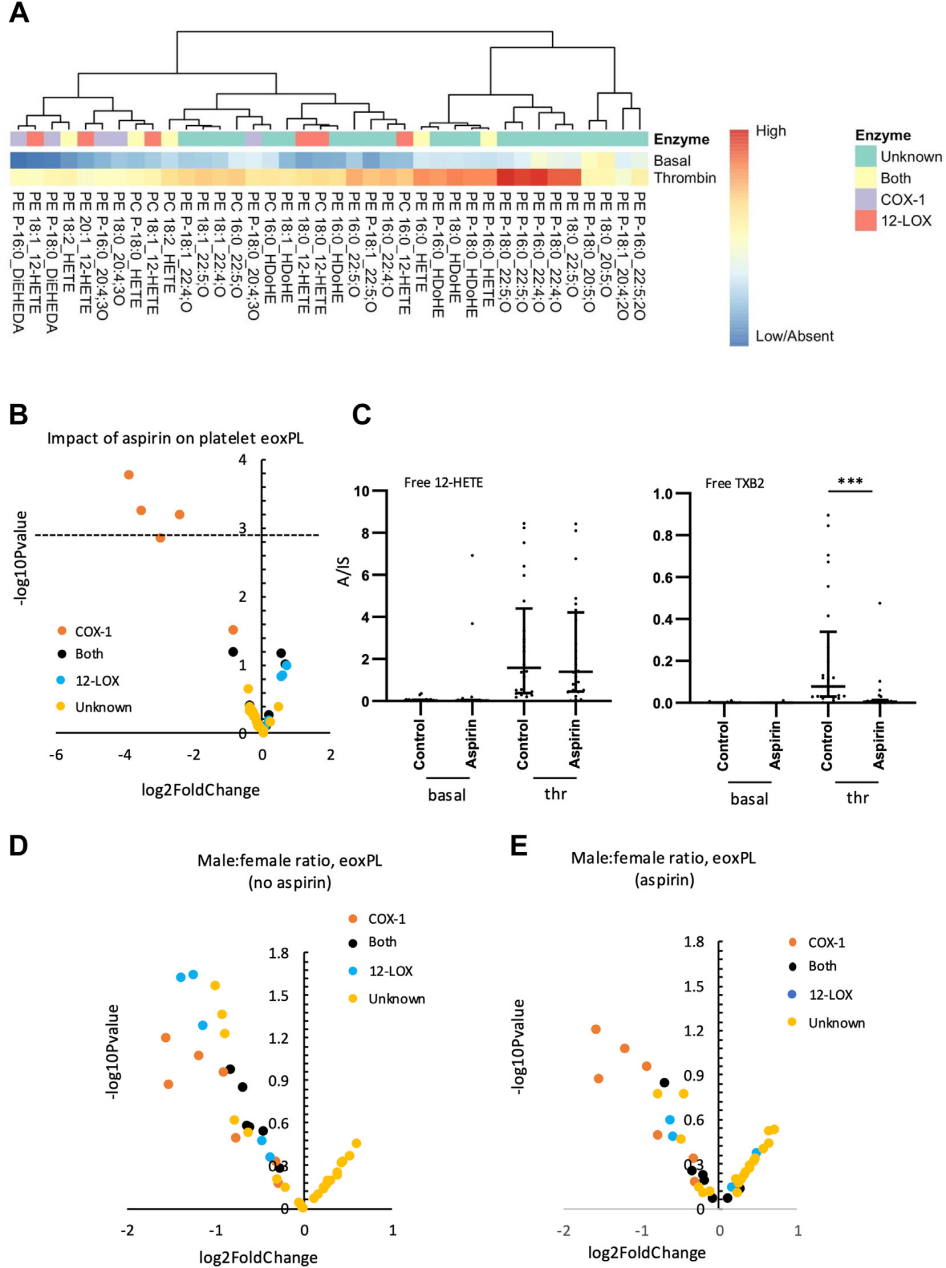
Thrombin stimulates generation of many eoxPL in platelets from healthy subjects, while aspirin supplementation reduces COX-derived eoxPL. Blood was collected from 28 healthy volunteers (14 males and 14 females) following an NSAID washout period, and platelets isolated and activated in vitro using thrombin, as outlined in Methods. Lipids were extracted from either resting or thrombin-activated platelets, and levels of 38 eoxPL profiled using LC/MS/MS, with analyte:internal standard (A/IS, cps) determined as outlined in Methods. Panel (A). *Heatmap showing generation of diverse eoxPL in healthy subjects*. Log10 values were calculated and plotted using Pheatmap in R as outlined in methods, with assignment to enzymatic sourced as outlined. Panel (B). Volcano plot showing impact of aspirin on platelet eoxPL. After providing a baseline sample, subjects were administered aspirin (75 mg/day) for a week, before repeat sampling of platelets. Data from the healthy cohort eoxPL were analyzed to generate log2fold change and -log10Pvalues (from paired *t*-tests), which were plotted with lipids labeled according to their enzymatic origin. Panel (C). *Free 12-HETE and TXB2*. These were measured in platelet lipid extracts using LC/MS/MS (n = 14, 50:50 male female) as outlined in Methods. Panels (D, E). Platelets from males generate lower levels of several eoxPL than females, in an aspirin-sensitive manner. Lipids were plotted on a volcano plot as -log10Pvalue and log2FoldChange, male:female, labeled by pathway of origin. Statistical significance was tested with paired *t*-tests (∗*P* < 0.05, ∗∗*P* < 0.01, ∗∗∗*P* < 0.001). The significance level shown on Panel (B) (dotted line, *P* < 0.05) was adjusted using Bonferroni correction for multiple comparisons. For panels (E, and F) no lipids were significantly different following Bonferroni correction. As data in Panel (C) were not normally distributed, Wilcoxon signed-rank test was used (n = 26, or 25 for basal or thrombin activated, respectively). eoxPL, enzymatically oxygenated phospholipid; COX, cyclooxygenase; ASCVD, atherosclerotic cardiovascular disease; NSAID, nonsteroidal antiinflammatory drug; HETE, hydroxyeicosatetraenoic acid.

To further test the impact of aspirin on platelet eoxPL, we turned to a healthy cohort. Previously, in platelets from three unrelated healthy human donors, we found that ∼100 eoxPL were generated acutely on thrombin activation (40). Some of these were already structurally characterized eoxPL, but many remain incompletely annotated with only partial structural information, eg specifying the chain length, number of rings/double bonds, and oxygenation (40). Taking this further, we expanded the study up to 28 subjects (50:50 male:female), to test the impact on eoxPL generation of (i) aspirin, (ii) gender, and (iii) seasonality. Subjects first provided a baseline blood sample following a nonsteroidal antiinflammatory drug washout, and the generation of the 48 most abundant eoxPL was measured (40). Lipids were putatively assigned as either COX-1 or 12-LOX-derived based on known sources of free oxylipins in platelets, eg 12-HETE (12-LOX), or DiEHEDA (COX-1) (45). We also considered oxylipins containing 20:4;3O as being likely prostaglandins, e.g. PGE_2_, which are known to be generated in eoxPL forms by platelets (46). Across the panel, 38 out of 48 lipids were significantly elevated on thrombin activation (Fig. 3A). Next, to analyze the impact of aspirin on thrombin stimulation of eoxPL generation, platelets were obtained after 1 week on 75 mg/day aspirin in the same individuals. Most lipids we had proposed to originate from COX-1 were significantly suppressed (Fig. 3B, orange markers). This data confirm the enzymatic origin of putative COX-1 derived eoxPL. The 12-HETE-containing eoxPL increased following aspirin supplementation, although this did not reach significance (Fig. 3B, blue markers). When analyzed for gender differences, the median diacyl 12-HETE-PL were consistently elevated in males following aspirin treatment, although this was only significant for PC 18:1_12-HETE (supplemental Fig. S8B, C). In contrast, aspirin had no discernible effect on diacy-12-HETE-PL generation in females (supplemental Fig. S9A). This small increase in diacyl-12-HETE-PL in males is similar to that seen in the clinical cohort, further supporting the idea that it relates to aspirin. Unfortunately, plasmalogen forms of 12-HETE-PL were below LOQ for these samples and could not be compared. The small elevations of diacyl-12-HETE-PL could result from either higher generation of free 12-HETE or increased rates of esterification of 12-HETE into lysoPL, by ACSL/LPLAT enzymes (33). To test this, levels of 12-HETE and TXB2 were analyzed in activated samples, however while TXB2 generation was suppressed around 95% by aspirin, there was no elevation in free 12-HETE for either males or females (Fig. 3C, supplemental Fig. S9B). This suggests that esterification of 12-HETE to form diacyl, but not plasmalogen-HETE-PL is enhanced by aspirin, supporting our observations in the cardiovascular cohort earlier.

Next, the influence of gender (in the absence of aspirin) was tested, and a trend toward reduced generation of several eoxPL in platelets from males was found, although following Bonferroni correction, these differences became not significant (Fig. 3D). The most affected lipids were either from LOX or unknown pathways, suggesting a lower overall thrombin response in males. Those that were reduced included two diacyl-12-HETE-PL (from LOX, PC 18:1a_12-HETE, and PC 16:0a_12-HETE) with two others being plasmalogen-PEs, PE 18:0p_20:5,O, and PE 18:1p_20:4,2O. None of these were impacted by aspirin in either males or females, indicating they are not generated by COX-1. However, when subjects were on aspirin, this small gender effect was attenuated (Fig. 3E). Gender differences in platelet reactivity were previously described and will be summarized in the Discussion.

### EoxPL from COX and LOX show seasonal variations which are abrogated by aspirin

**Fig. 4:**
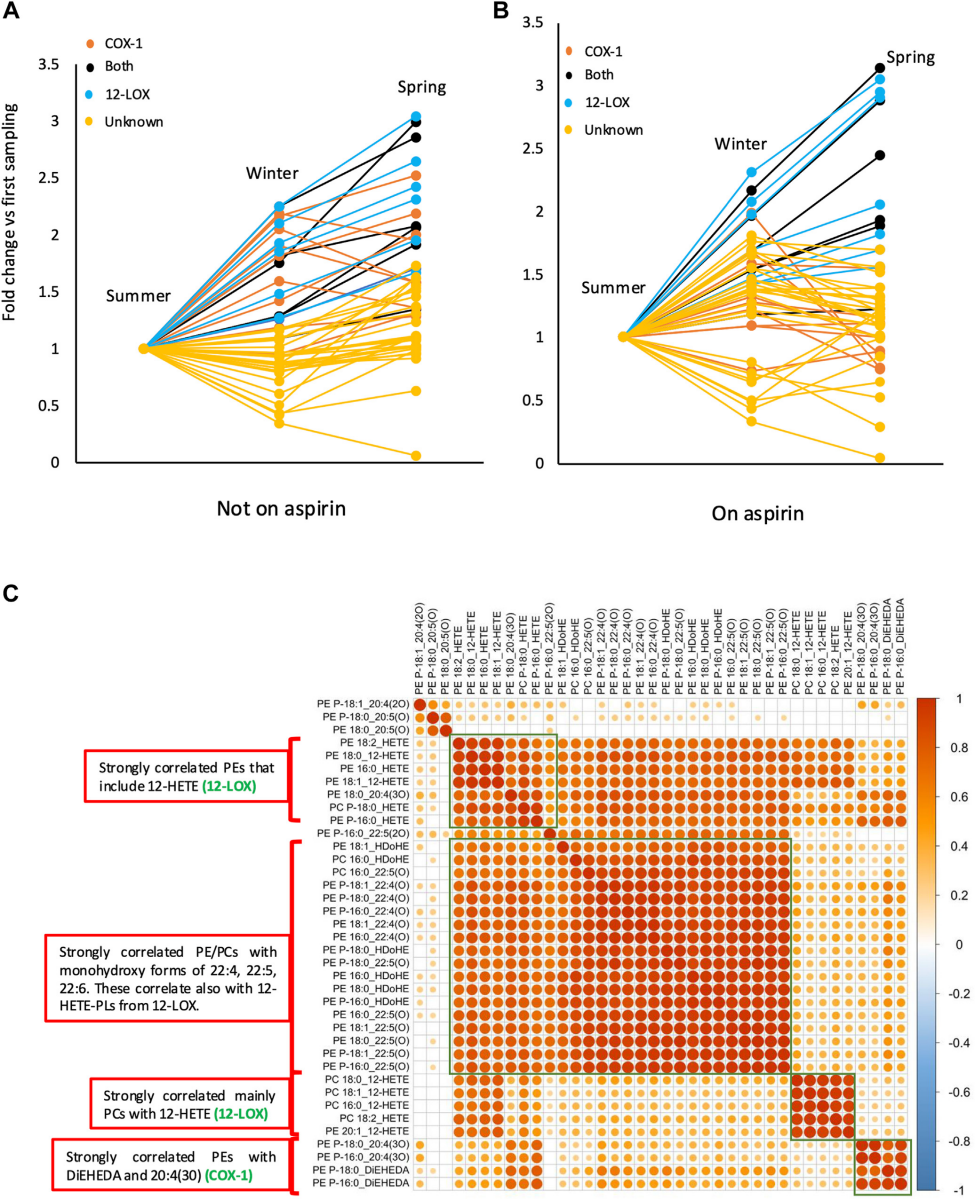
eoxPL generated by COX show seasonal variation that is attenuated by aspirin supplementation. Healthy volunteers (n = 14) donated platelets before and after 1 week aspirin (75 mg/day) supplementation. The donation protocol was repeated after 2–3 months, to generate repeat samples for each volunteer. Lipids were extracted from platelets (2 × 10^8^) basally or following thrombin activation (0.2 U/ml) as in Methods. Extracts were analyzed using LC/MS/MS, calculating A/IS values for each lipid at each time point, with volunteers either off (Panel A) or on (Panel B) aspirin. Summer: June-Aug, Winter: Dec-Feb, Spring: March-May. Panel (C). Pearson’s correlation coefficients demonstrate clustering of eoxPL by Sn2 fatty acyl composition and headgroups. Pearson’s correlations were calculated and plotted as shown. eoxPL, enzymatically oxygenated phospholipid; COX, cyclooxygenase.

The interindividual variability of platelet eoxPL, when repeatedly sampled over time, is unknown. To test this, a subgroup of healthy volunteers (7 males and 7 females) returned at two subsequent two monthly intervals to provide additional samples pre- and post-aspirin. When subjects were not on aspirin, generation of eoxPL from COX/LOX were up to 2.5-3-fold higher the following spring, a relatively modest fluctuation (Fig. 4A). In contrast, most lipids assigned as “unknown” did not show any seasonal variation (Fig. 4A). When subjects were on aspirin, the increase in COX-derived lipids in spring was not seen since their generation was reduced overall (Fig. 4B), although for LOX-derived eoxPL the seasonal trend remained, while as before “unknowns” were largely unaffected.

### EoxPL correlate based on Sn-2 oxylipin composition

To assess biochemical relatedness of eoxPL molecular species, specifically, whether lipids showed behavior consistent with common biochemical/enzymatic origins, correlation plots were generated using the data from the first sampling time point (n = 28, basal, thrombin, ± aspirin supplementation). Here, groupings aligned with *Sn*-2 fatty acyl oxylipin structure strongly (Fig. 4C). For example, PEs containing HETEs, HDOHEs, or other monohydroxy lipids clustered together in groups. Briefly, 12-HETE-PC formed a separate cluster, indicating influence of the headgroup on biochemical behavior. COX-1 eoxPL correlated closely but were very distinct from other lipids, most likely due to the selective impact of aspirin. There were no negative correlations since all lipids either correlated positively or not at all. These data suggest that eoxPL are generated in groups based on common enzymatic pathways, and this will be explored next.

### LPCAT3 generates diacyl-eoxPL from 12-HETE in platelets, but not those from COX-1, nonenzymatic, or longer chain n3 PUFA-derived eoxPL

Aspirin supplementation in the clinical cohort demonstrated a complex regulation of platelet eoxPL, with 12-LOX-derived diacyl forms being selectively elevated, while COX-1 derived forms were reduced (Fig. 1). These data, along with our correlation analysis (Fig. 4C) suggest a complex biochemical network whereby structurally related eoxPL maybe formed via specific isoforms of ACSL/LPLATs via Lands cycle (33). However, to date, no information are available on which isoforms generate eoxPL in primary cells.

**Fig. 5:**
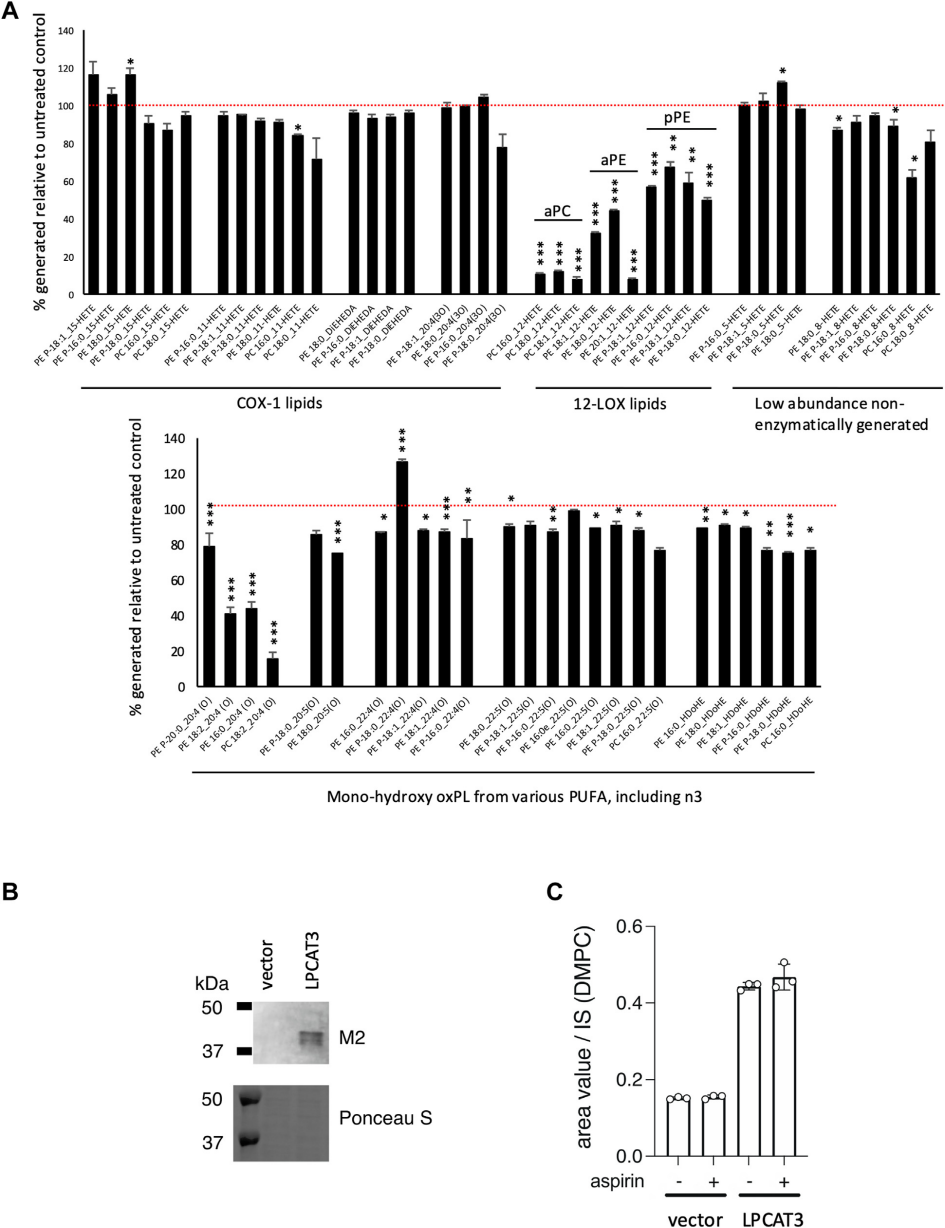
LPCAT3 plays a critical role in 12-LOX-derived eoxPL formation, and its activity is not directly affected by aspirin. Panel (A). *Platelet eoxPL from 12-HETE requires LPCAT3, but not eoxPL generated by COX-1 or from longer chain* n*3 PUFA*. Washed platelets were activated with thrombin following a 30 min incubation with 10 μM (*R*)-HTS-3, as described in Methods, then lipids extracted and analyzed using LC/MS/MS. The experiment was repeated with three independent donor isolates, and a representative is shown (n = 3 replicates, mean ± SEM), Students *t* test, ∗*P* < 0.05, ∗∗*P* < 0.01, ∗∗∗*P* < 0.005. Panel (B): Flag tagged LPCAT3 was detected using M2 Flag antibody in Western blot as per Methods. Panel (C): *Cell*-free LPCAT assay was performed as in Methods using 20 μM C16:0 d31LPC and 20 μM C20:4-CoA as substrates with or without 1 mM aspirin. Data are shown by the mean ± SD of triplicate measurements. IS is an internal standard (dimyristoyl-PC, DMPC, PC 14:0/14:0). eoxPL, enzymatically oxygenated phospholipid; COX, cyclooxygenase; LOX, lipoxygenase; HETE, hydroxyeicosatetraenoic acid; LPCAT3, lysophosphatidylcholine acyltransferase 3.

A strong candidate in platelets for supporting esterification of HETEs is LPCAT3 (LPLAT12 (34), MBOAT5) since this is specific for 18:2 and 20:4 and can use lysoPC, lysoPE, and lysoPS as substrates (34, 47, 48, 49, 50). Mice deficient in LPCAT3 do not survive to adulthood, so obtaining their platelets was not possible. An LPCAT3 inhibitor, *(R*)-HTS-3, recently became available, so this was tested in human platelets from three unrelated healthy donors (37). This compound has been shown to be inactive toward the related enzymes, LPCAT1 and LPIAT1, indicating some selectivity (37). Not only HETE-PL, but a wider group of previously identified platelet eoxPL were measured using LC/MS/MS, including additional COX-1 products, monohydroxy forms of longer chain PUFA (20:5, 22:3, 22:4, and 22:5) and nonenzymatic platelet products (5- and 8-HETE-PL) (40). The data suggest that LPCAT3 is required for generation of diacyl-12-HETE-PC and PE (Fig. 5A). However, plasmalogen-PE forms were less inhibited by *(R*)-HTS-3. Strikingly, COX-1-derived eoxPL and those containing longer chain n3 PUFA were not or only slightly affected (Fig. 5A). These data suggest that LPCAT3 selectively esterifies 12-HETE into eoxPL in platelets, showing a strong preference for acyl forms of lysoPL over plasmalogens. Esterification of 11- or 15-HETEs into PL was not mediated by LPCAT3, despite their structural similarity to 12-HETE.

Since in vivo aspirin supplementation elevated the levels of eoxPL that were shown here to be generated in an LPCAT3-dependent manner, we next examined the effect of aspirin on LPCAT3 activity directly. Here, human LPCAT3 was tested in the presence of 1 mM aspirin. Microsomal fractions of CHO cells overexpressing human LPCAT3 were used as an enzyme source. Aspirin did not affect LPCAT activity of LPCAT3 (Fig. 5B, C). Together, these data suggest that aspirin does not directly affect LPCAT3 and that the selective regulation of diacyl-12-HETE-PL generation by aspirin may instead relate to changes in LPCAT3 expression, expression or activity of upstream ACSLs, or levels of substrates available.

### Lipidomics demonstrates higher levels of platelet eoxPL in human arterial limb and coronary (STEMI), than carotid thrombi

Next, to study in vivo generated eoxPL, arterial thrombi from patients were analyzed, comparing three anatomical sites: carotid, limb, and STEMI. Clots surgically obtained from patients were homogenized, lipids extracted, and HETE-PL measured using LC/MS/MS, followed by hydrolysis of fatty acyls and analysis of HETEs for chirality to confirm enzymatic origin. Briefly, 12-HETE from platelet 12-LOX, 15-HETE from leukocyte 15-LOX, and 5-HETE from neutrophil 5-LOX should be primarily *S*, while 15-HETEs from COX-1 can be either *R* or *S* (51, 52). The 11-HETE generated by COX-1 is predominantly *R* (51, 53).

**Fig. 6:**
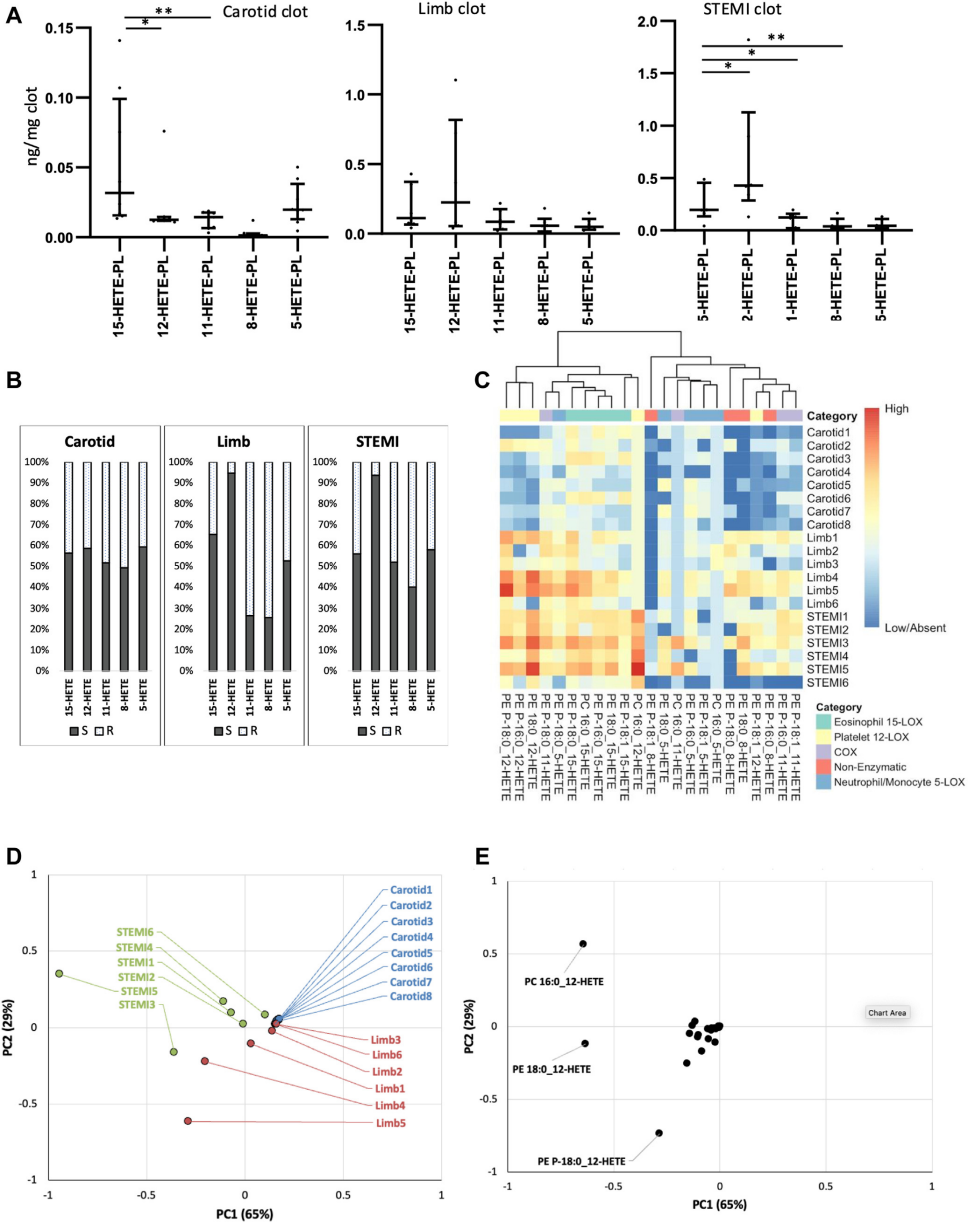
eoxPL profiles of limb and STEMI clots show a strong platelet signature and carotid clots are more abundant in white cell isoforms. Panel (A): The profile of arterial thrombi eoxPL shows site-specific signatures. Arterial thrombi were extracted, snap-frozen on dry ice, and frozen at −80°C from patients undergoing angioplasty for ST-elevation myocardial infarction (STEMI, n = 6), carotid endarterectomy (carotid n = 8) or peripheral vascular embolectomy (limb n = 6). Clots were homogenized and lipids extracted as in Methods, then analyzed using LC/MS/MS, quantified, and normalized by tissue weight (ng lipid/100 mg clot). Panel (B): Chiral analysis confirms the platelet signature of limb and STEMI clots based on high S/R ratio of 12-HETE. Clots (n = 3 each) lipid extracts were hydrolyzed, then analyzed using chiral LC/MS/MS as in Methods. Panel (C): The eoxPL dataset plotted as a heatmap shows site specific features. A heatmap with hierarchical clustering was plotted using the pheatmap R package after log10 normalization of data. Panel (D): Principal component analysis of arterial thrombi by HETE-PL profile shows clustering of clots with common lipids. eoxPL data were analyzed using the prcomp function in R (v version 3.5.3) Panel (E): The corresponding loading plot shows the variables most influential in explaining the difference between samples (each dot represents a lipid). Statistical significance was tested with Mann-Whitney-Wilcoxon test for pairwise comparison after Kruskal-Wallis test for nonparametric analysis of variance on rank (∗*P* < 0.05, ∗∗*P* < 0.01, ∗∗∗*P* < 0.001). eoxPL, enzymatically oxygenated phospholipid; HETE, hydroxyeicosatetraenoic acid.

Although the thrombi were from a small group of patients with differences due to age, demographics, and clinical background, there were clear eoxPL signatures seen that related to anatomical site. In carotid clots, 15-, 11-, and 5-HETE-PL were the prominent eoxPL with lower levels of 12- and 8-HETEs. This suggested white cells as the main source of eoxPL (Fig. 6A). Chiral analysis of carotid clot lipids showed that 12-, 15-, and 5-HETEs were all slightly more prominent as *S* isomer forms (55%–60%) while both 8- and 11-HETEs were around 50% *S/R*. Coupled with the higher levels of 15-HETE-PL versus 12- or 8-HETE-PL, we suggest that 15-HETE-PL originates from both leukocyte 15-LOX (*S*) and COX (*S/R*), while some of the 5-HETE-PL is from neutrophil 5-LOX (*S*) (Fig. 6B). The other HETE-PL, 12-, 8-, and 11- may be nonenzymatically derived. There was notable absence of 12-HETE-PL in carotid clots, suggesting little or no platelet involvement (Fig. 6A). In contrast, STEMI and limb clots were characterized by higher levels of 12-, followed by 15-, and 11-HETE-PL, then lower amounts of 5- and 8-HETE-PL. This indicated a platelet signature, largely dominated by 12-LOX (12-HETE-PL) with some COX-1-derived eoxPL (11-, 15-HETE-PL). As confirmation, limb and STEMI clots contained around 95% 12*S*-HETE (Fig. 6B). In limb clots, 11-HETE was around 75% the *R* isomer, consistent with COX-1, although in STEMI, it was around 50% (Fig. 6B). Subsequently, 8-HETE in limb and STEMI clots was around 75% or 60% *R* (Fig. 6B). Although nonenzymatic oxidation would predict 50% S/R for 8-HETE, a similar predominance of the *R* enantiomer in serum from clotted blood was previously shown (53, 54), and 8*R*-HETE is a known side product of platelet 12-LOX (55). In limb and STEMI clots, 5-HETE-PL were relatively low in comparison to other positional isomers and were around 50%–60% *S* isomer (Fig. 6B). Thus, they may be primarily nonenzymatic, with a small involvement of neutrophil 5-LOX. Notably, clots contain large amounts of hemoglobin which could directly contribute to nonenzymatic oxidation, either in vivo or during lipid extraction, despite inclusion of antioxidants and metal chelators.

The full dataset for all lipids is shown as a heatmap (Fig. 6C). The relatively higher abundance of 12- and 15-HETE-PL in STEMI and limb clots, and the contribution of individual lipids can be seen. Next, a principal component analysis was undertaken, to confirm which lipids contributed most strongly to the phenotype of the clot types. This demonstrated separation of the STEMI and limb embolectomy samples in the PC1 direction (responsible for 65% of the variance) (Fig. 6D). The loadings plot demonstrates that the HETE-PL lipids most responsible for this separation in PC1 are three containing 12-HETE, namely two diacyl and one plasmalogen (Fig. 6E).

## Discussion

How procoagulant membranes of blood cells are altered in cardiovascular disease is poorly understood, along with how they contribute to thrombotic risk, and also their involvement in formation of human clots in situ. Previously, eoxPL were shown to be elevated in blood cells from patients with venous thrombosis, and are procoagulant in vitro and in vivo (56). However, up to now, their formation in ASCVD has not been investigated. Herein, using lipidomics, we characterized eoxPL in human ASCVD in blood cells and retrieved clots, as well as in platelets from a healthy cohort. In ASCVD patients, platelet eoxPL generation showed complex changes that primarily were associated with aspirin supplementation, findings which were then confirmed in a healthy cohort. Complex impacts of aspirin on eoxPL generation were uncovered that can be summarized as follows: (i) inhibition of COX-1 derived eoxPL generation, (ii) elevation of Lands cycle-dependent diacyl-12-HETE-PL generation in platelets, via LPCAT3, (iii) abrogation of gender and seasonal impacts on eoxPL generation in healthy volunteers. These data show that aspirin’s impact on bleeding/thrombosis may move beyond platelet aggregation blockade, into modulation of membrane-dependent coagulation. How eoxPL modulation by aspirin impacts thrombotic risk via coagulation in humans in health and disease warrants further study.

Our study found that LPCAT3 mediates diacyl-12-HETE-PE/PC generation in platelets, providing the first direct evidence of a specific acylation enzyme forming eoxPL. Up to now, the particular Lands cycle enzymes catalyzing oxylipin esterification have remained unknown. The expression and activity of LPCAT3, and other LPAT isoforms in platelets is currently unknown and requires further study. The lack of involvement of LPCAT3 in esterification of 11- or 15-HETEs generated by COX-1 suggests there maybe compartmentalization of 12-HETE-PL biosynthetic enzymes within platelets, specifically phospholipaseA_2_, 12-LOX, ACSL, and LPCAT3. This is further supported by our previous observations that exogenously-added 12-HETE-*d8* is not esterified into eoxPL following acute platelet activation to form 12-HETE-*d8*-PE, in place of endogenously-generated 12-HETE (10). Also, the lack of esterification of monohydroxy-oxylipins from longer chain unsaturated PUFA by LPCAT3 is consistent with the known substrate preference of this enzyme for 20:4.

Low-dose aspirin increased platelet generation of diacyl 12-HETE-PL. Although this could be due to increased availability of arachidonate substrate when COX-1 is inhibited (57), free 12-HETE generation by platelets was not elevated by aspirin in the healthy cohort, and only eoxPL which we found are formed by LPCAT3 were affected. This indicates that generation of eoxPL is also controlled at the level of esterification, suggesting LPCAT3 is somehow modulated by aspirin indirectly. Further studies are needed to determine the mechanism. In contrast, inhibition of COX-derived eoxPL by aspirin is expected to be due to direct inhibition of the enzyme. No difference in eoxPL generation was seen for the small number of patients on P2Y12 inhibitors. This receptor is the target of ADP activation of platelets, and we previously found that ADP is unable to induce 12-HETE generation by platelets providing a potential explanation for this finding (58).

As 12-HETE-PL are the most quantitatively abundant positional isomers of HETE-PL, the overall impact of aspirin appears to result in higher total levels of platelet eoxPL in platelets from patients taking this drug. Importantly, resting platelets contain little or no eoxPL, with these lipids being generated following agonist challenge. This means that, in vivo, while a patient is otherwise healthy, eoxPL in the circulation should be very low, unless a challenge occurs, eg through trauma, infection, or plaque rupture. Where 12-LOX-derived lipids are elevated by aspirin, they could potentially antagonize the antithrombotic impact of thromboxane A2 (TXA_2_) inhibition, through promoting the procoagulant impact of the platelet membrane. The clinical impact of aspirin has always been considered to be primarily via its antiplatelet effects, not via modulation of coagulation per se. In support, washed platelets from patients on aspirin therapy still mount a procoagulant phenotype in vitro (59, 60, 61). However, this does not exclude that eoxPL may influence coagulation under the conditions under which they are generated within the complex milieu of a thrombus in vivo. In line with this, a strong signature of platelet derived eoxPL was seen in retrieved human STEMI and limb clots, although it is not known at what stage of thrombus formation they were generated (eg early or late). Agreeing with the proposed role of these lipids in driving arterial coagulation in vivo, *Alox15*^*−/−*^ mice, which lack many eoxPL, generated smaller thrombi in an arterial model of AAAs (15), and higher levels of eoxPL are associated with prothrombotic autoimmunity (8). Furthermore, we previously showed that both *Alox15*^*−/−*^ and *Alox12*^*−/−*^ mice form smaller venous thrombi in vivo and have a hemostatic defect that can be corrected using eoxPL administration (15).

Leukocyte generation of eoxPL was also modulated in patients with arterial disease, specifically increased neutrophil 5-HETE-PL were detected, combined with moderate reductions in other isoforms. Decreased 11- and 15-HETE-PE could result from inhibition of white cell COX-1 by aspirin; however, due to the small patient numbers a clear effect was not obvious, and also, 15-HETE-PL in leukocytes can also come from eosinophil 15-LOX which is not sensitive to aspirin (14). In white blood cell (WBC) samples, a likely source of 12-HETE-PL is platelet-leukocyte aggregates, since human WBC do not generally express 12-LOX. These are elevated in ASCVD, and aspirin can dampen their formation (62, 63). Overall, the detailed reasons for WBC eoxPL changing in disease are not clear and require further study.

To test our hypothesis that aspirin regulated eoxPL in ASCVD, we next analyzed samples from a healthy cohort. Here, complex gender and seasonal differences in platelet eoxPL formation were seen. First, platelets from females tended to mount a stronger response to thrombin in relation to eoxPL formation. This finding agrees with numerous previous studies showing that platelets from females aggregate more robustly to several agonists in vitro (64, 65, 66). The rather small gender difference was largely abrogated by aspirin supplementation, suggesting that it may somewhat rely on secondary activation triggered by TXA_2_. A second gender difference was seen for LPCAT3-dependent formation of diacyl 12-HETE-PL, which tended to be elevated in males by aspirin, but not females. This mirrored the significant elevations in males in the clinical cohort and showing a gender-specific effect of aspirin on a subgroup of eoxPL by human platelets. How hormones regulate the procoagulant membrane, specifically via LPCAT3, is currently unknown, despite the well-known gender bias for thrombotic risk in men, which increases in women post menopause. We note that in our clinical cohort, the small number of females would be expected to be post menopausal, while in the health cohort, they were all younger than age 50. Last, the relative expression of LOX, COX, and LPATs in males versus females is unknown.

Unexpectedly, while most eoxPL were relatively stable across the three sampling periods, COX-1 and LOX derived eoxPL steadily increased throughout, peaking in spring. Although only a relatively small increase of 1.5–3 fold, it was consistently observed for ∼15 individual lipid species. The seasonal change for COX-1-derived eoxPL was blunted by aspirin. The basis of this fluctuation is unknown, and we found no reports of seasonal variation in platelet activities in the literature. Unfortunately, a fourth sample per patient was not obtained during the study period and so eoxPL generation could not be followed further. Although samples were in storage for different times, we do not expect this to have selectively led to changes in only some eoxPL versus others as storage time was overall less than a year.

We previously used chiral chromatography to evidence the direct enzymatic involvement of LOX enzymes in generating platelet and Th2 macrophage eoxPL (10, 11). Taking this further, here, limb or STEMI clots were characterized by a strong platelet eoxPL signature, while white cell or nonenzymatic eoxPL dominated the composition of carotid thrombi. Although these clots were formed in vivo under flow, the content of the clot could be influenced by other anatomical/pathological factors. For instance, carotid clot may be contaminated by significant amount of plaque lipid relative to clot. This is due to the nature of the surgical extraction technique which may also explain the lower total amounts of oxPL. Consistent with this, a predominance of 15-HETE-PL was seen in carotid samples which may originate from macrophages/foam cells in plaque lesions (67). Free 15-HETE has previously been reported as a component of human carotid plaques, but was deemed to be nonenzymatically generated (68). Our chiral analysis agrees with this, since it indicated relatively similar proportions of *S* and *R* stereoisomers in carotid plaque. This is consistent with studies examining the enantiomeric composition of hydroxyoctodecadienoic acids within advanced atherosclerotic plaques (69). The physiological age of the analyzed clots may also contribute differences. STEMI clots were retrieved within a short time from formation (<3 h) compared to carotid clots (days/weeks). Thus, the 12*S*-HETE-PL dominant profile in STEMI clots may relate to an acute burst of platelet activation in the first few hours of thrombosis, compared to the carotid clots where time has passed since formation which allows for clot metabolism to take place by the generation of an organized fibrin scaffold, modification to the cellular components and possible PL degradation (70, 71, 72). This could also contribute to higher observed amounts of HETE-PL seen owing due to a level of inflammatory cell activation in the acute phase (73). Clots collected from limb embolectomies varied in retrieval time (“age”), and this may explain the mixed picture observed in the multivariate analysis where despite an overall similar profile to STEMI, at least two of the “oldest” clots were behaving in a way closer to carotid clots. Other factors that may explain the variability include interindividual variability, comorbidity profile, medications, or lifestyle choices. However, the current sample size is not sufficiently powered to investigate this in depth.

### Study limitations

While cells from patients with ACS demonstrated lipidomic and biological differences, it is not possible to determine causality since it is not possible to predict when an ACS event will occur (74). Furthermore, the use of A23187 (a nonphysiological agonist of leukocytes) was to assess the ability and capacity of WBC to generate more eoxPL on stimulation compared with resting states, and given its nonphysiological nature, it would not be valid to use this to test how differences in HETE-PL on the surface of stimulated WBC affect coagulation in health and disease. Moreover, it is difficult to disentangle the contribution of free 15-HETE and esterified 15-HETE to thrombosis in vivo given the ability of 12/15-LOX to directly esterify AA-containing PL and the untested potential of LPCAT3 inhibitors to be used in-vivo (11). This will form part of future plans for follow-on studies which could also include creating conditional or megakaryocyte specific knockouts to test the role of LPCAT3. Finally, the cross-sectional design of the clinical cohort and the absence of longitudinal sampling time points (particularly for the acute phase of ACS) may be confounded by interindividual variations which may reduce statistical power. This, alongside the relatively small sample size, and low levels of some lipids makes further validation and replication in a larger cohort desirable.

## Conclusions

In summary, procoagulant eoxPL generation is altered in platelets and leukocytes from patients with ASCVD, potentially contributing to thrombotic risk. Aspirin is the most widely prescribed drug globally for secondary vascular prevention (75, 76), with its primary effect being inhibition of the platelet lipid, thromboxane A2 (TXA_2_) (77). Clinically, aspirin is generally used as an antiplatelet agent in arterial disease, versus anticoagulants like warfarin which predominate clinically in venous thrombosis (78). Here, aspirin but not P2Y12 inhibitors had a complex and selective impact on eoxPL generation in both health and disease, thus its influence on the procoagulant membrane needs to be considered. The identification of LPCAT3 as a regulator of the procoagulant membrane which is influenced by aspirin identifies a potential target which deserves further characterization in relation to thrombotic risk.

## Data availability

All processed data are available as a supplementary file with the manuscript. Raw data are available on reasonable request: o-donnellvb@cardiff.ac.uk or prottym3@cardiff.ac.uk.

## Supplemental data

This article contains supplemental data.

## Conflict of interest

P. C. receives research funding from CSL Behring, Haemonetics Corp, Werfen, and consultancy from CSL Behring. The other authors declare that they have no conflicts of interest with the contents of this article.
