# Supplementary material for: Aspirin modulates generation of procoagulant phospholipids in cardiovascular disease, by regulating LPCAT3
Source: J Lipid Res. 2024 Dec 12;66(1):100727. doi: 10.1016/j.jlr.2024.100727 (PMC11754521; doi:10.1016/j.jlr.2024.100727)
Supplement: Supplementary Figures, Legends and Tables [file mmc1.pdf]

Supplementary Figures, Legends and Tables.

Supplementary Figure 1: Example chromatograms for lipids measured in the study. LC/MS/MS was conducted as outlined in Methods.

Supplementary Figure 2: CAD and ACS platelets have reduced COX-1 generated 11-HETE-PL but similar amounts of 8-HETE-PE and 12-LOX generated plasmalogen 12-HETE-PL species. Platelets were isolated, activated and lipid extracts then generated and analyzed as described in the legend to Figure 1 and Methods. ACS (n=24), CAD (n=19), RF (n=23), HC (n=24). Panel A shows 8-HETE-PL. Panels B-D show three plasmalogen 12-HETE-PL lipids. Panels E-J show six individual 11-HETE-PL. Statistical significance was tested with Mann-Whitney-Wilcoxon test for pairwise comparison after Kruskal-Wallis test for non-parametric analysis of variance on rank (*p <0.05, ** p <0.01, *** p <0.001).

Supplementary Figure 3: Aspirin reduces COX-1 generated 15-HETE-PL species and increases 12-LOX generated diacyl 12-HETE-PL in thrombin-activated platelets in ASCVD*. Panels A-E: Impact of disease on generation of 15-HETE-PL lipids*. Platelets were isolated, activated and lipid extracts then generated and analyzed as described in the legend to Figure 1 and Methods. ACS (n=24), CAD (n=19), RF (n=23), HC (n=24). Statistical significance was tested with Mann-Whitney-Wilcoxon test for pairwise comparison after Kruskal-Wallis test for non-parametric analysis of variance on rank (*p <0.05, ** p <0.01, *** p <0.001). *Panel F. Aspirin supplementation is associated with elevated levels of diacyl but not plasmalogen 12-HETE-PL in platelets from CAD, and RF patients' platelets, respectively.* CAD: n = 5, and 14 for no aspirin, and aspirin, respectively, RF: n = 3, and 20 for no aspirin, and aspirin, respectively. Statistical significance was tested with Mann-Whitney-Wilcoxon test for pairwise comparison (*p <0.05, ** p <0.01, *** p <0.001).

Supplementary Figure 4: Aspirin increases 12-LOX generated diacyl 12-HETE-PL in thrombin-activated platelets from CAD and RF males*.* Platelets were isolated, activated and lipid extracts then generated and analyzed as described in the legend to Figure 1 and Methods. n = 8, and 20 for no aspirin, and aspirin, respectively. Statistical significance was tested with Mann-Whitney-Wilcoxon test for pairwise comparison (* p <0.05, ** p <0.01, *** p <0.001).

Supplementary Figure 5: Aspirin reduces COX-1 generated 11- and 15-HETE-PL species in thrombin-activated platelets in ASCVD*.* Platelets were isolated, activated and lipid extracts then generated and analyzed as described in the legend to Figure 1 and Methods. CAD: n = 5, and 14 for no aspirin, and aspirin, respectively, RF: n = 3, and 20 for no aspirin, and aspirin, respectively. *Panel A. CAD, 11-HETE-PLs, Panel B, RF,11-HETE-PL, Panel C, RF, 15-HETE-PE, Panel D, RF, 15-HETE-PE*. Statistical significance was tested with Mann-Whitney-Wilcoxon test for pairwise comparison (* p <0.05, ** p <0.01, *** p <0.001).

Supplementary Figure 6: 12-HETE-PL generation is reduced in activated leukocytes from patients with ASCVD while 5-HETE-PL are similar. Leukocytes were isolated, activated and lipid extracts then generated and analyzed as described in the legend to Figure 2 and Methods. *Panel A. 5-HETE levels were relatively similar. Panel B. 12-HETE-PLs generated by activated leukocytes are reduced in disease*. ACS (n=24), CAD (n=19), RF (n=23), HC (n=24). Statistical significance was tested with Mann-Whitney-Wilcoxon test for pairwise comparison after Kruskal-Wallis test for non-parametric analysis of variance on rank (*p <0.05, ** p <0.01, *** p <0.001).

Supplementary Figure 7: Ionophore-activated leukocytes generate lower levels of 11- and 5-HETE-PL in patients with ASCVD. Leukocytes were isolated, activated and lipid extracts then generated and analyzed as described in the legend to Figure 2 and Methods. *Panel A. 11-HETE-PL levels were reduced in platelets from patients with arterial disease.*

Panel B. 15-HETE-PLs generated by activated leukocytes are reduced in disease ACS (n=24), CAD (n=19), RF (n=23), HC (n=24). Statistical significance was tested with Mann-Whitney-Wilcoxon test for pairwise comparison after Kruskal-Wallis test for non-parametric analysis of variance on rank (*p <0.05, ** p <0.01, *** p <0.001).

Supplementary Figure 8: Generation of 15-HETE-PL by WBC in RF and CAD patients is increased by aspirin, while diacyl 12-HETE-PL from LOX are generally increased in male healthy donor platelets administered aspirin. *Panel A. The impact of aspirin on HETE-PL generation by leukocytes from CAD and RF patients.* Leukocytes were isolated, activated and lipid extracts then generated and analyzed as described in the legend to Figure 2 and Methods. (CAD: n = 5, and 14 for no aspirin, and aspirin, respectively, RF: n = 3, and 20 for no aspirin, and aspirin, respectively). Statistical significance was tested with Mann-Whitney-Wilcoxon test for pairwise comparison (* p <0.05, ** p <0.01, *** p <0.001). *Panel B. Plots showing diacyl 12-HETE-PL molecular species in platelets from males are slightly elevated by aspirin supplementation*. Lipids were extracted and analyzed using LC/MS/MS as outlined in Methods. Statistical significance was tested with paired T-tests (*p <0.05, ** p <0.01, *** p <0.001).

Supplementary Figure 9: Aspirin supplementation does not influence generation of diacyl 12-HETE-PL (females) or free 12-HETE (males or females). Platelets were isolated from 28 healthy volunteers (n = 14 male, 14 female), activated, and lipids extracted and analyzed using LC/MS/MS, as outlined in the legend for Figure 3 and Methods. *Panel A. Plots showing 12-HETE-PL molecular species in platelets which were not elevated by aspirin supplementation*. Lipids were extracted and analyzed using LC/MS/MS as outlined in Methods. *Panel B. Plots showing that aspirin prevents TXB_2 generation in males and females while having no impact on free 12-HETE*. Lipids from thrombin-activated platelets were extracted and analyzed using LC/MS/MS as outlined in Methods. Statistical significance was tested with paired T-tests (*p <0.05, ** p <0.01, *** p <0.001). For Panel B Wilcoxon Signed-Rank Test was used (n = 31, or 12 for male or female, respectively).

Supplementary Figure 1

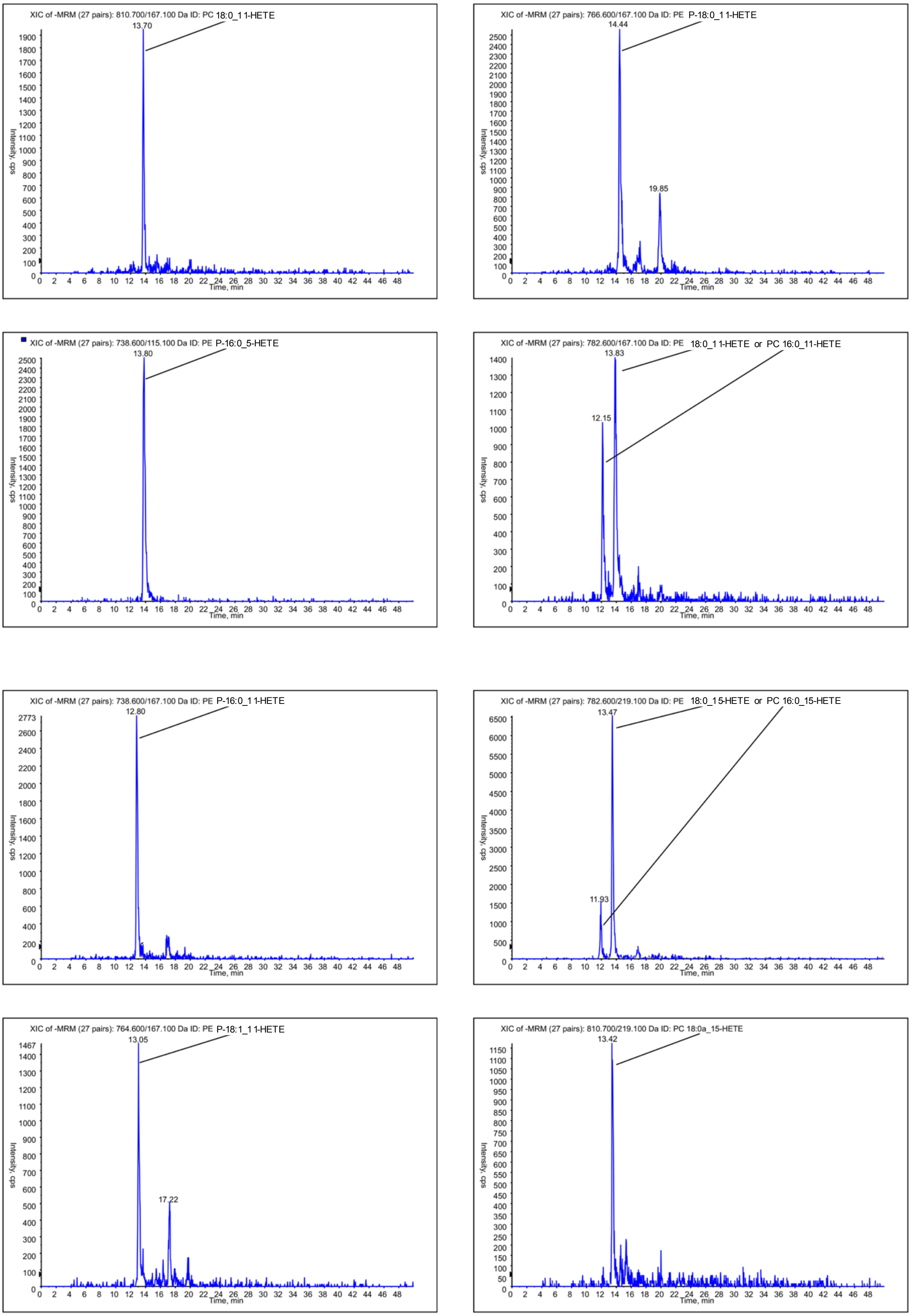

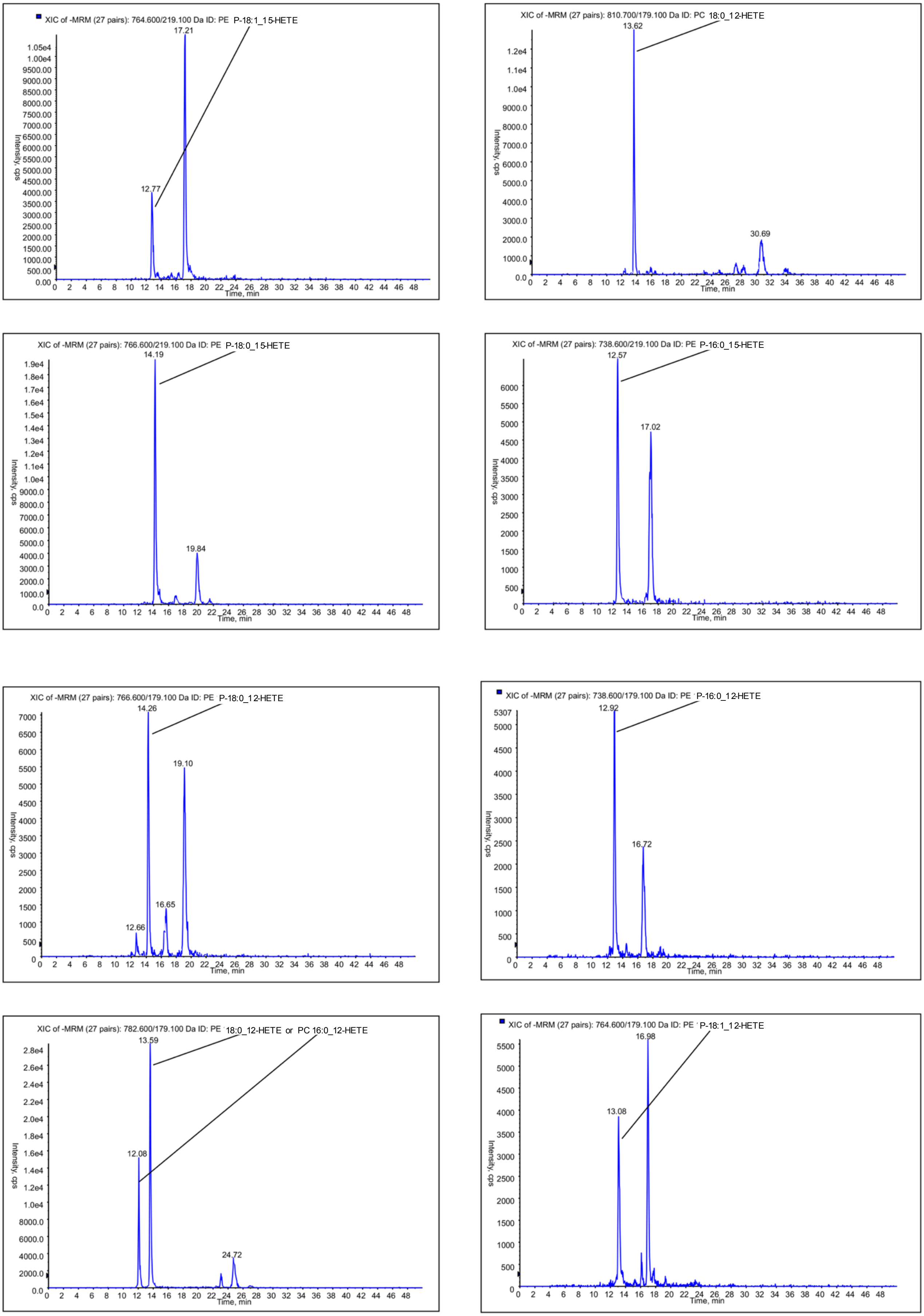
XIC of -MRM (27 pairs): 764.600/219.100 Da ID: PE P-18:1_15-HETE
17.21
12.77
Intensity, cps
Time, min
XIC of -MRM (27 pairs): 810.700/179.100 Da ID: PC 18:0_12-HETE
13.62
30.69
Intensity, cps
Time, min
XIC of -MRM (27 pairs): 766.600/219.100 Da ID: PE P-18:0_15-HETE
14.19
19.84
Intensity, cps
Time, min
XIC of -MRM (27 pairs): 738.600/219.100 Da ID: PE P-16:0_15-HETE
12.57
17.02
Intensity, cps
Time, min
XIC of -MRM (27 pairs): 766.600/179.100 Da ID: PE P-18:0_12-HETE
14.26
19.10
16.65
12.66
Intensity, cps
Time, min
XIC of -MRM (27 pairs): 738.600/179.100 Da ID: PE P-16:0_12-HETE
12.92
16.72
Intensity, cps
Time, min
XIC of -MRM (27 pairs): 782.600/179.100 Da ID: PE 18:0_12-HETE or PC 16:0_12-HETE
13.59
12.08
24.72
Intensity, cps
Time, min
XIC of -MRM (27 pairs): 764.600/179.100 Da ID: PE P-18:1_12-HETE
16.98
13.08
Intensity, cps
Time, min

Supplementary Figure 1, contd

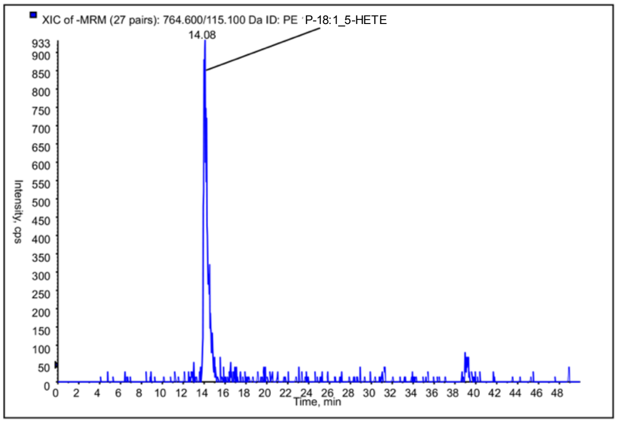

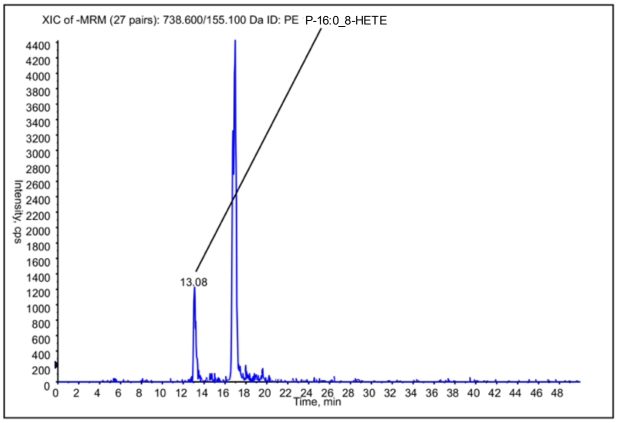

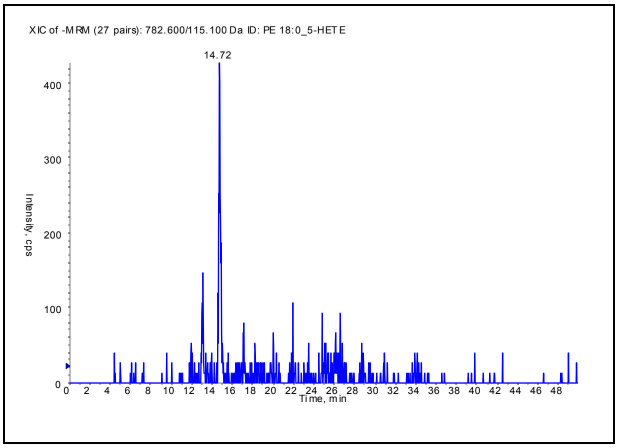

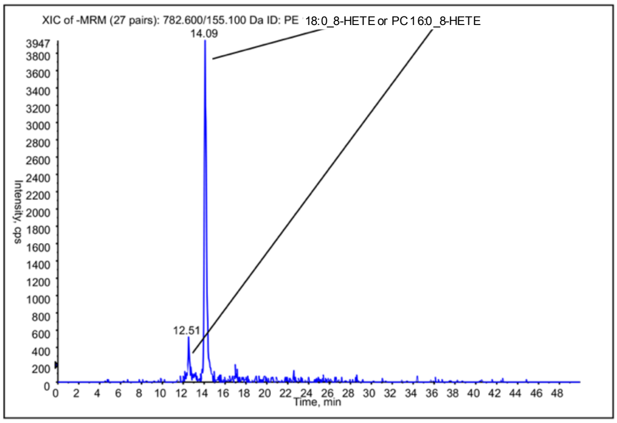

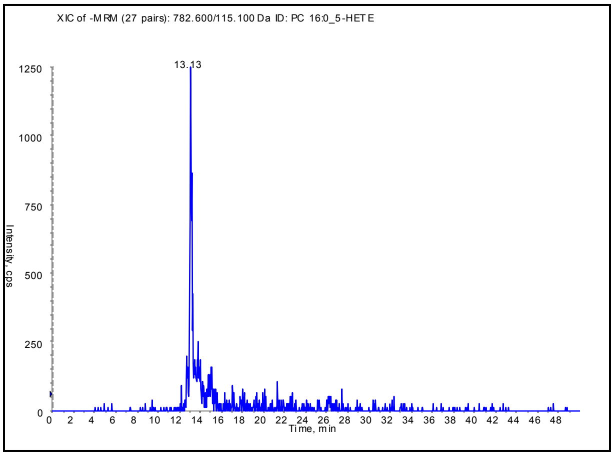

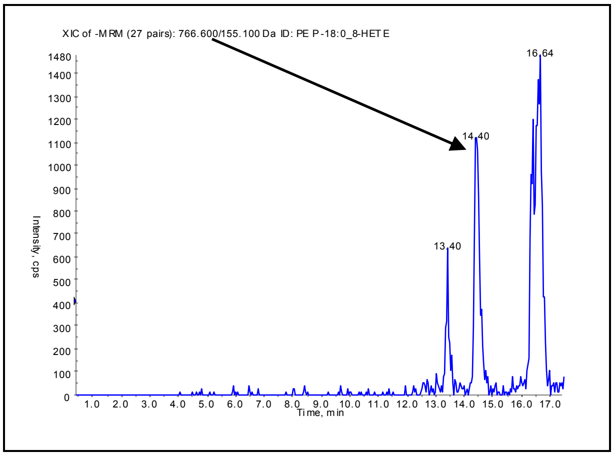

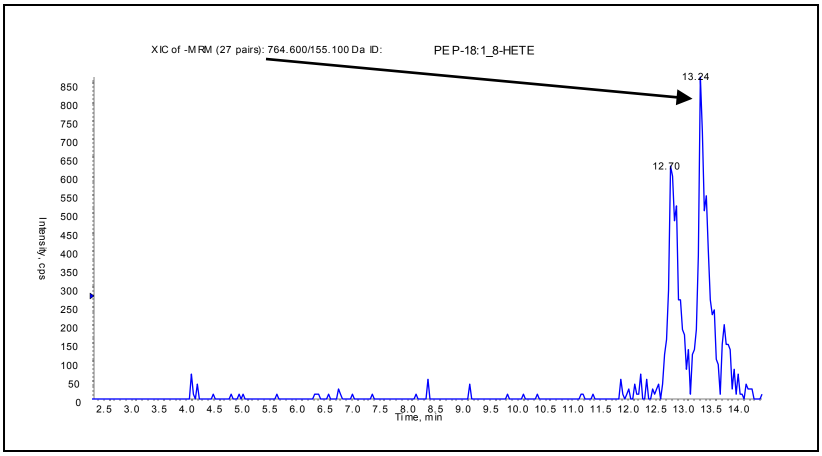

Supplementary Figure 2

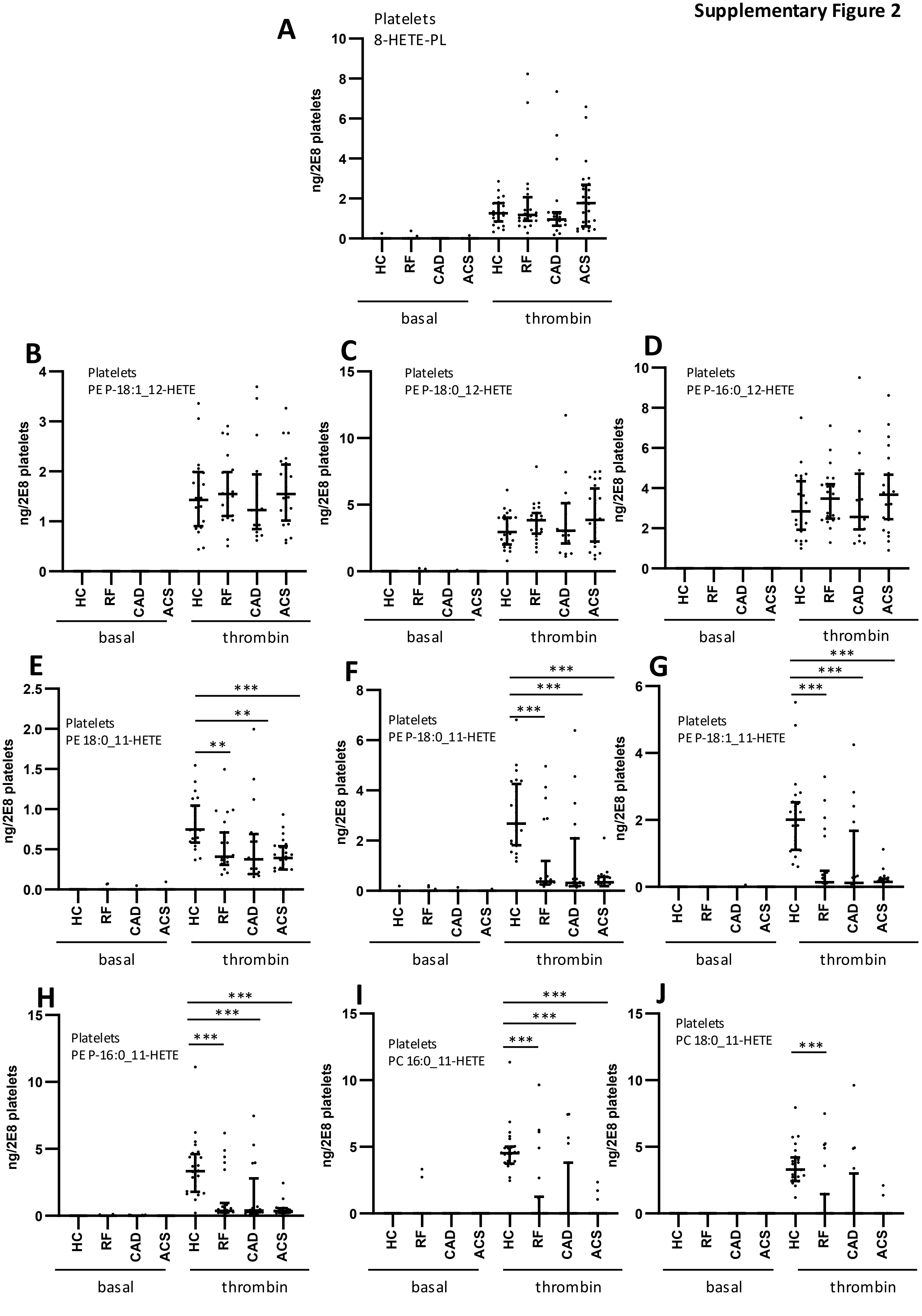

Supplementary Figure 3

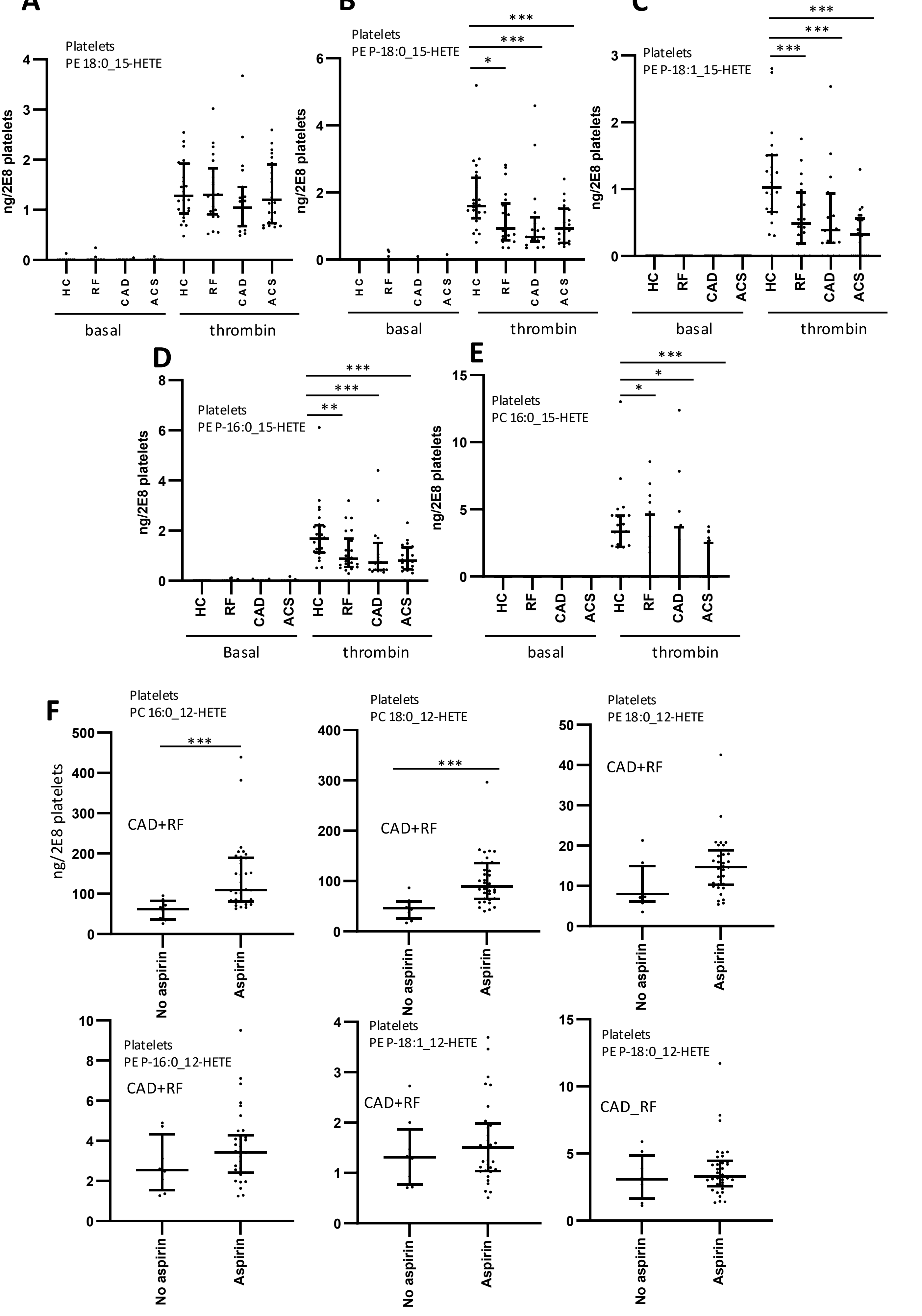

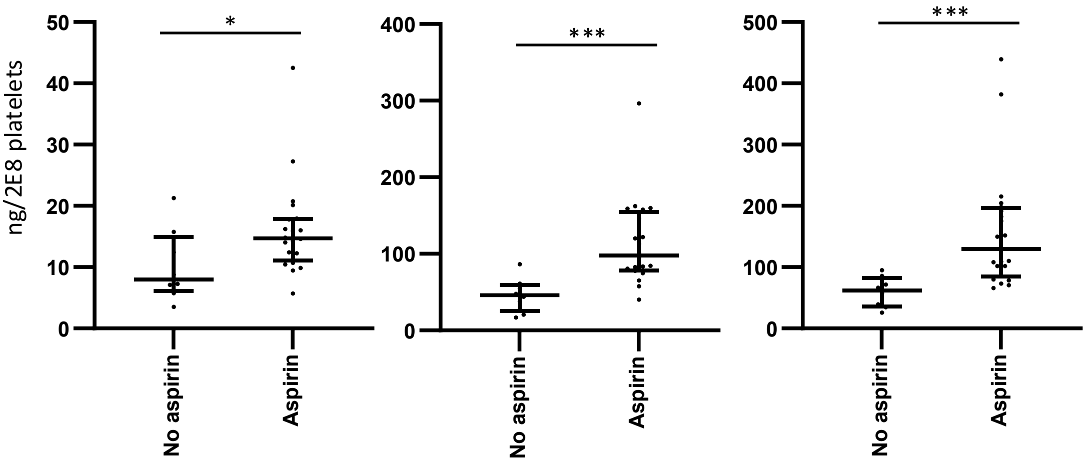

Platelets
PE 18:0_12-HETE
RF + CAD groups

Platelets
PC 18:0_12-HETE
RF + CAD groups

Platelets
PC 16:0_12-HETE
RF + CAD groups

Supplementary Figure 5

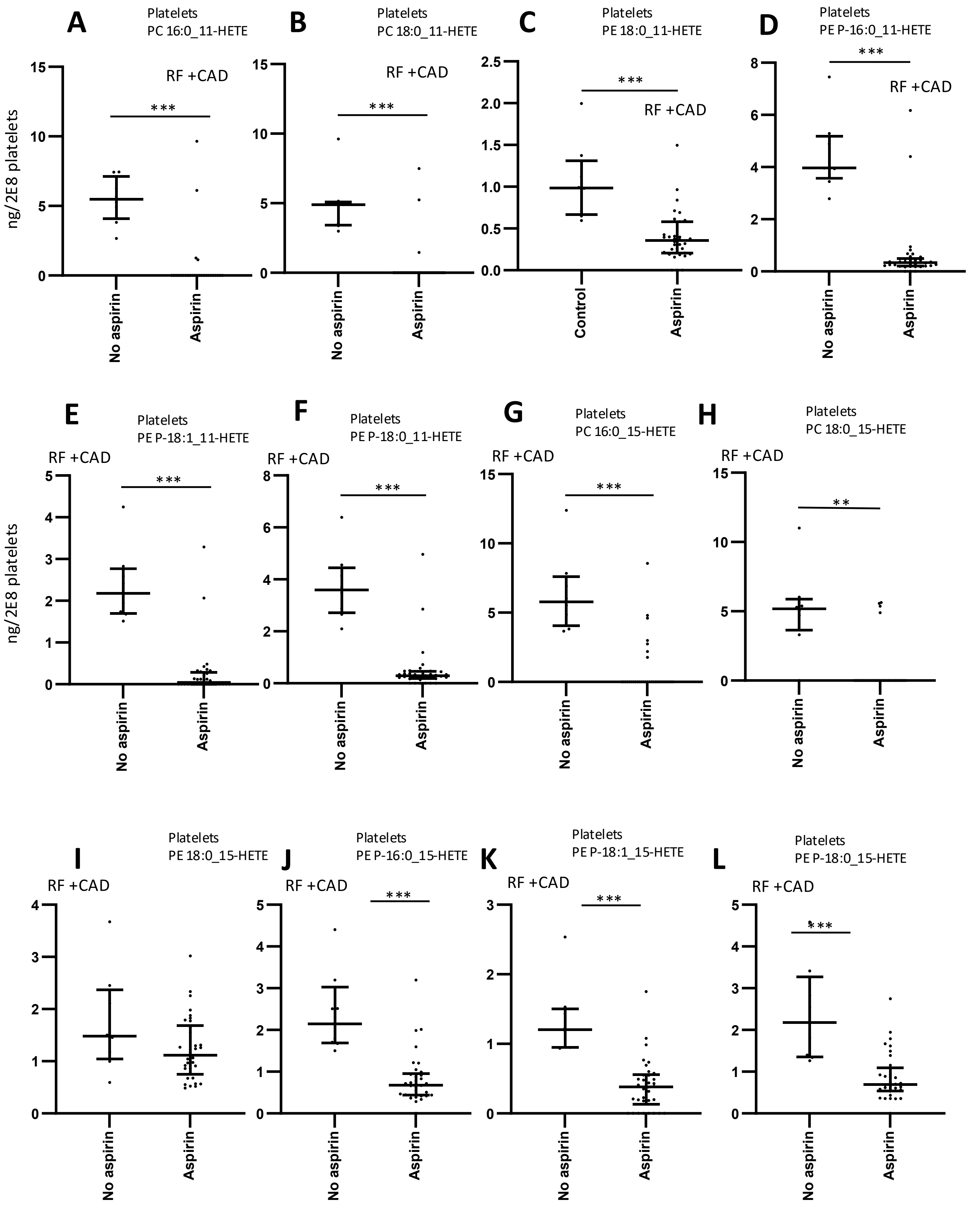

Supplementary Figure 6

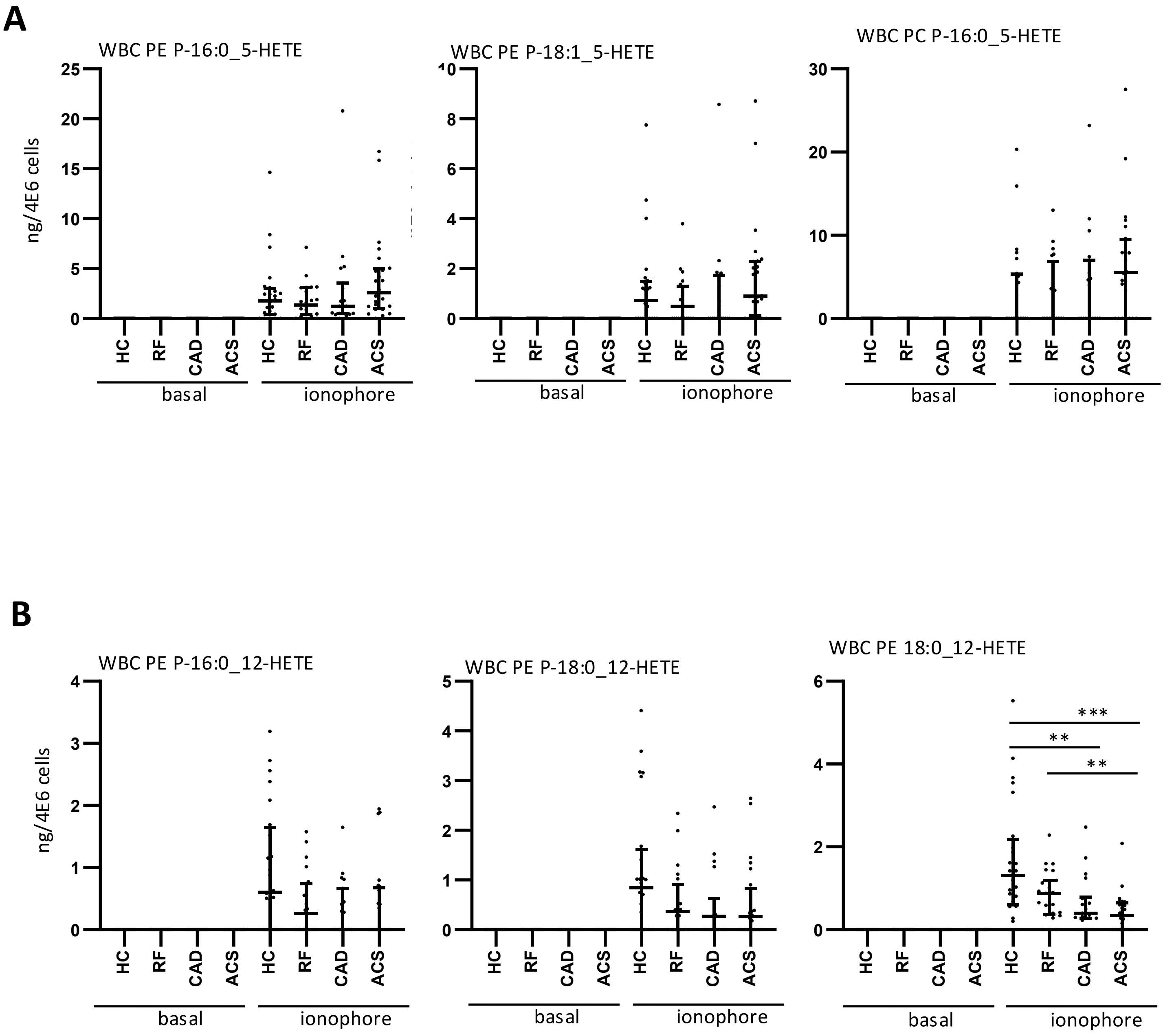

Supplementary Figure 7

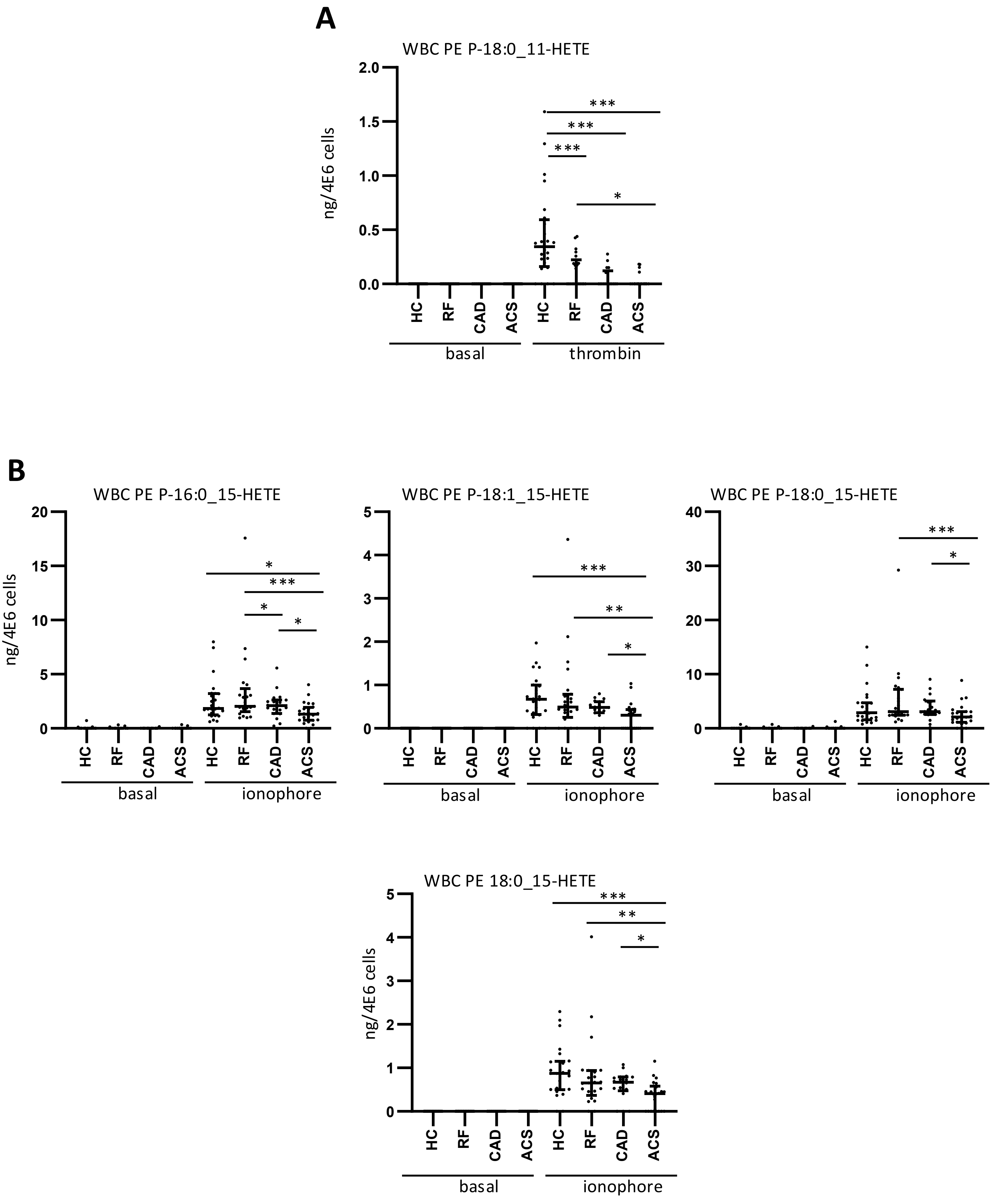

Supplementary Figure 8

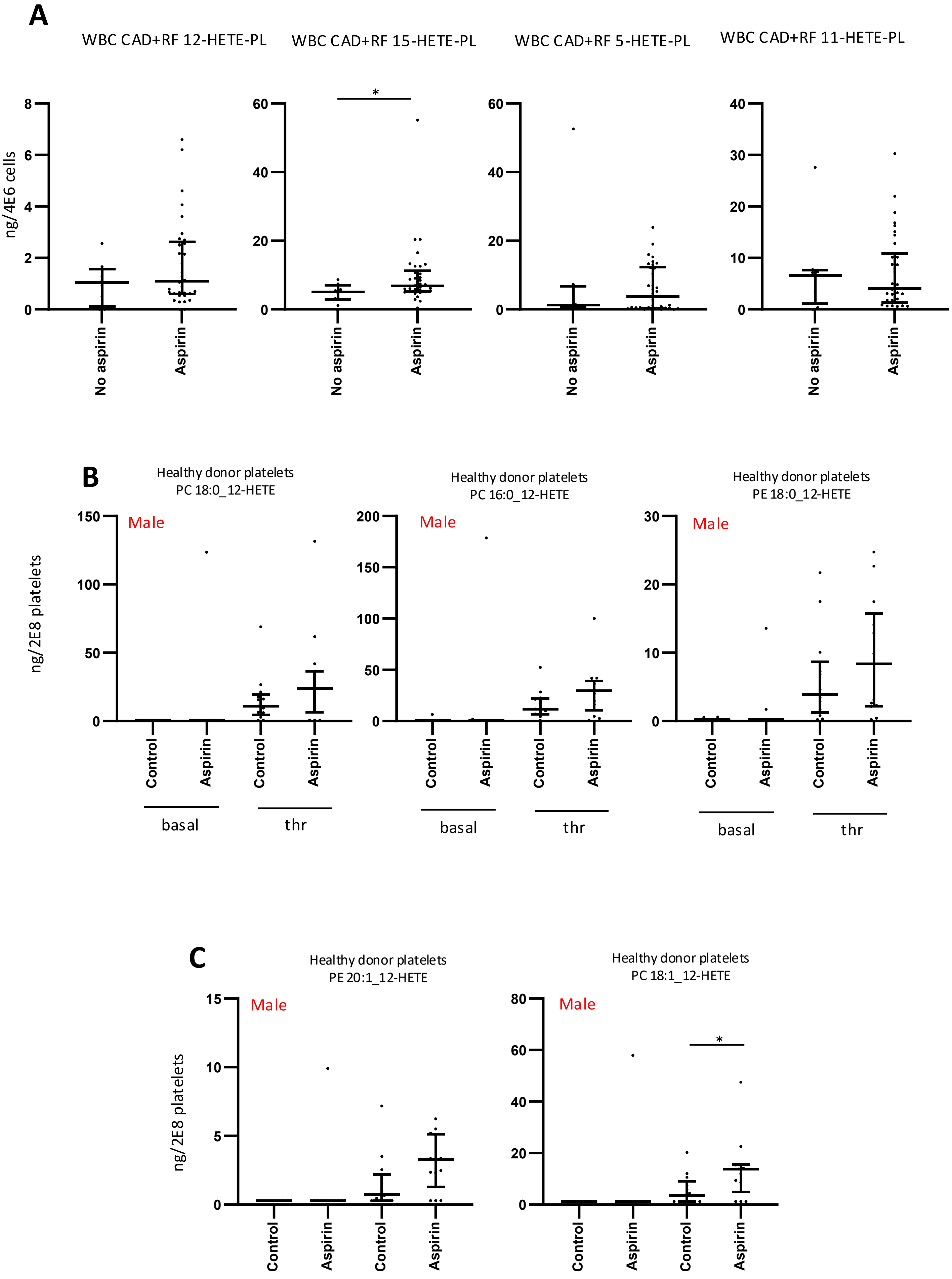

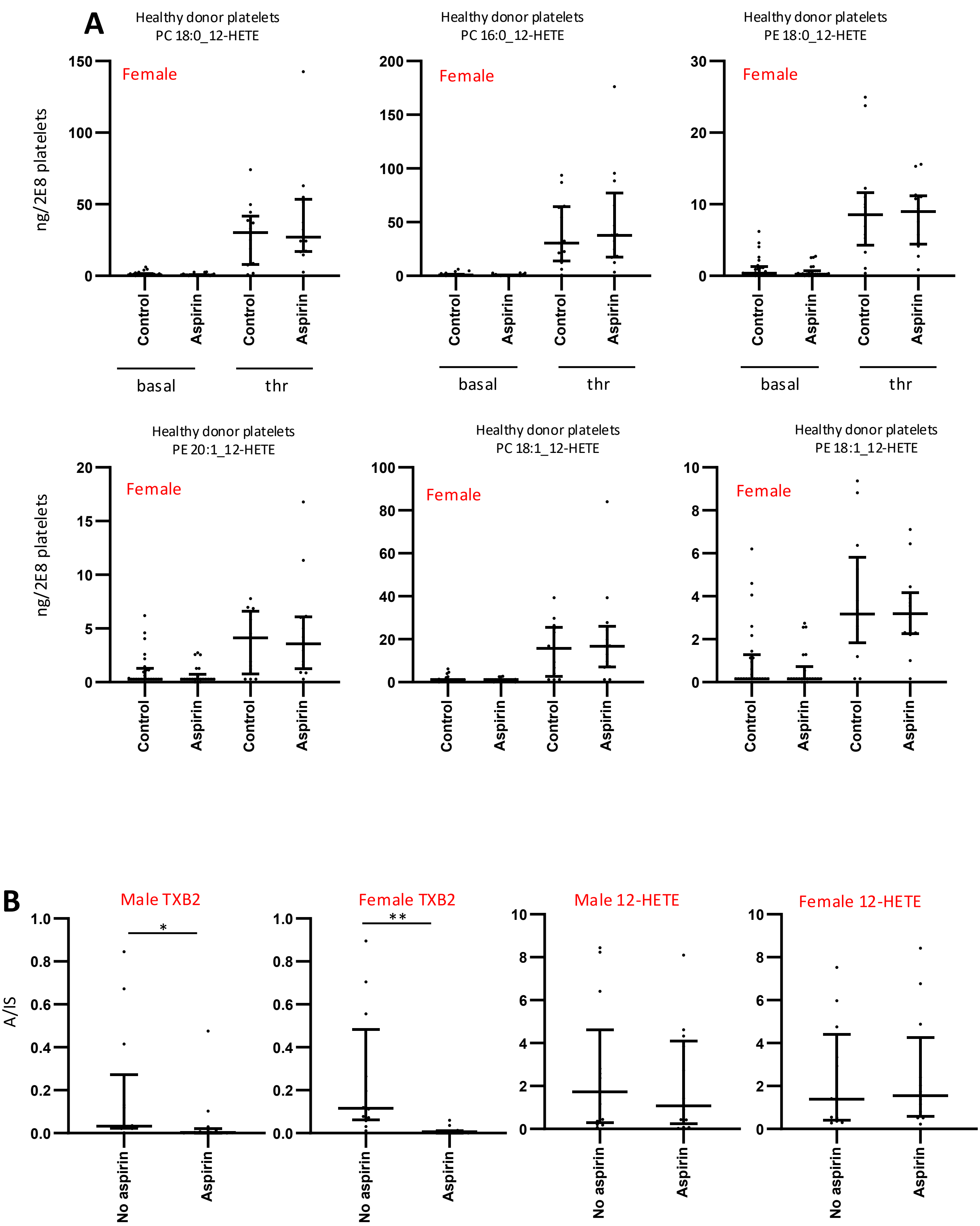
A
Healthy donor platelets
PC 18:0_12-HETE
Female
ng/2E8 platelets
150
100
50
0
Control
Aspirin
Control
Aspirin
basal
thr
Healthy donor platelets
PC 16:0_12-HETE
Female
200
150
100
50
0
basal
thr
Healthy donor platelets
PE 18:0_12-HETE
Female
30
20
10
0
basal
thr
Healthy donor platelets
PE 20:1_12-HETE
Female
20
15
10
5
0
Healthy donor platelets
PC 18:1_12-HETE
Female
100
80
60
40
20
0
Healthy donor platelets
PE 18:1_12-HETE
Female
10
8
6
4
2
0
B
Male TXB2
*
A/IS
1.0
0.8
0.6
0.4
0.2
0.0
No aspirin
Aspirin
Female TXB2
**
Male 12-HETE
Female 12-HETE
10
8
6
4
2
0

Supplementary Table 1: Baseline clinical characteristics of patients recruited in the clinical cohort. (WCC: white cell count, RBC: red blood cell count, P2Y12 inhibitors: clopidogrel, prasugrel or ticagrelor, CKD: chronic kidney disease, SD: standard deviation, p-value tests: Fisher exact (categorical) or Kruskal-Wallis (continuous), p-value comparators: all clinical groups (age, gender) or all except HC for other variables)

**Variable**	**Healthy control (HC) (n=24)**	**Risk-factor matched (RF) (n=23)**	**Significant coronary artery disease (CAD) (n=19)**	**Acute coronary syndrome (ACS) (n=24)**	**p**
Age, Mean ± SD	64.92 ± 10.79	61.43 ± 8.16	64.53 ± 9.65	64.67 ± 9.88	0.473
Gender, male n (%)	14 (58.3)	12 (52.2)	16 (84.2)	17 (70.8)	0.133
Creatinine μmol/L, Mean ± SD	-	77.57 ± 12.60	88.39 ± 31.95	84.79 ± 18.64	0.318
Hemoglobin g/dL, Mean ± SD	-	146.04 ± 13.03	138.24 ± 17.95	144.46 ± 18.88	0.380
Platelets x 10^9/L, Mean ± SD	-	250.26 ± 44.01	261.94 ± 56.08	271.50 ± 82.72	0.869
WCC x 10^9/L, Mean ± SD	-	6.96 ± 1.57	7.92 ± 1.92	9.28 ± 2.90	0.011
Neutrophils	-	4.33 ± 1.17	5.02 ± 1.32	6.51 ± 2.49	0.003
Eosinophils	-	0.19 ± 0.09	0.25 ± 0.12	0.11 ± 0.08	<0.001
Basophils	-	0.02 ± 0.04	0.03 ± 0.05	0.00 ± 0.02	0.098
Lymphocytes	-	1.82 ± 0.68	1.89 ± 0.70	1.85 ± 0.71	0.818
Monocytes	-	0.57 ± 0.19	0.68 ± 0.21	0.73 ± 0.32	0.150
RBC x 10^{12}/L, Mean ± SD	-	4.80 ± 0.42	4.14 ± 1.26	4.64 ± 0.54	0.090
Aspirin use (%)	0 (0)	20 (87)	14 (73.7)	24 (100)	0.017
P2Y12 inhibitor use (%)	0 (0)	3 (13)	6 (31.6)	24 (100)	<0.001
Anticoagulant use (%)	0 (0)	3 (13)	0 (0)	24 (100)	-
Statin use (%)	0 (0)	15 (65.2)	15 (78.9)	19 (79.2)	0.532
Hypertension (%)	0 (0)	13 (56.5)	13 (68.4)	11 (45.8)	0.350
Diabetes (%)	0 (0)	7 (30.4)	3 (15.8)	6 (25)	0.554
Smoker (%)	0 (0)	7 (30.4)	9 (47.4)	13 (54.2)	0.244
CKD (%)	0 (0)	0 (0)	3 (15.8)	3 (12.5)	-

Supplementary Table 2: Baseline clinical characteristics of extracted clots from patients with atherothrombosis undergoing surgical/interventional clot retrieval. (P2Y12 inhibitors: clopidogrel, prasugrel or ticagrelor, CKD: chronic kidney disease)

Clot ID	Tissue origin	Age	Gender	Aspirin	P2Y12 inhibitor	Anticoagulant	Statin	Hypertension	Diabetes	Smoker	CKD
STEMI1	Left anterior descending artery	66-70	Male	Yes	Yes	Warfarin	No	Yes	No	Never	No
STEMI2	Left anterior descending artery	56-60	Male	Yes	Yes	Heparin	No	No	No	Never	No
STEMI3	Right coronary artery	56-60	Male	Yes	Yes	Heparin	Yes	Yes	No	Ex	No
STEMI4	Left anterior descending artery	81-85	Male	Yes	Yes	Heparin	Yes	Yes	No	Never	Yes
STEMI5	Left anterior descending artery	56-60	Male	Yes	Yes	Heparin	Yes	Yes	Yes	Never	No
STEMI6	Right coronary artery	76-80	Female	Yes	Yes	Heparin	No	No	No	Ex	No
Carotid1	Left Carotid artery	71-75	Female	No	No	No	No	No	No	Ex	No
Carotid2	Left Carotid artery	66-70	Male	Yes	Yes	No	Yes	Yes	Yes	Ex	No
Carotid3	Right Carotid artery	71-75	Female	No	Yes	No	Yes	No	No	Never	No
Carotid4	Left Carotid artery	61-65	Male	Yes	Yes	Warfarin	Yes	Yes	No	Never	No
Carotid5	Right Carotid artery	76-80	Male	No	Yes	No	Yes	Yes	No	Never	No
Carotid6	Left Carotid artery	66-70	Male	No	Yes	No	Yes	Yes	No	Ex	No
Carotid7	Right Carotid artery	61-65	Male	Yes	Yes	No	Yes	Yes	No	Never	No
Carotid8	Left Carotid artery	56-60	Female	Yes	Yes	No	Yes	No	No	Current	No
Limb1	Right Femoral Embolectomy	56-60	Male	Yes	Yes	Heparin	No	No	No	Never	No
Limb2	Left Popliteal (Pop-Ped bypass)	46-50	Male	No	Yes	No	Yes	No	No	Current	No
Limb3	Left Femoral (Fem-Pop bypass)	61-65	Female	Yes	No	No	No	Yes	Yes	Ex	No
Limb4	Left Popliteal Below-Knee amputation	56-60	Male	No	No	Rivaroxaban	No	Yes	Yes	Ex	No
Limb5	Right Brachial Embolectomy	76-80	Female	Yes	No	Heparin	No	Yes	No	Never	No
Limb6	Right Popliteal (Fem-Pop bypass with embolectomy)	61-65	Male	No	Yes	No	Yes	No	No	Ex	No

Supplementary Table 3: Baseline biochemical and hematological characteristics of extracted clots from patients with atherothrombosis undergoing surgical/interventional clot retrieval.

Clot age is the estimated time from diagnosis (clot formation/plaque disruption) to retrieval time. (Creat: serum creatinine μmol/L, Hb: hemoglobin g/dL, Plt: platelet count x 10^9/L, WCC: total white cell count x 10^9/L, Neutrophils/Eosinophils/Basophils/Lymphocytes/Monocytes: respective count x 10^9/L, RBC: red blood cell count x 10^{12}/L)

Clot ID	Clot age	Creat	Hb	Plt	WCC	Neutrophils	Eosinophils	Basophils	Lymphocytes	Mono-	RBC
STEMI1	<3hr	137	172	168	12.1	10.7	0	0	0.9	0.5	5.3
STEMI2	<3hr	73	128	287	16.2	14.7	0.2	0	0.6	0.6	4.3
STEMI3	<3hr	96	149	301	7.1	5.4	0.1	0	1.1	0.4	4.5
STEMI4	<3hr	183	137	219	17	14.7	0.1	0	0.9	1.2	4.8
STEMI5	<3hr	92	132	283	14	12.5	0	0	0.5	1.5	4.4
STEMI6	<3hr	83	120	202	8.1	6.1	0	0	1.4	0.5	3.9
Carotid1	14 days	65	128	208	5.8	3.8	0.1	0.1	1.3	0.6	3.4
Carotid2	20 days	77	130	233	10.7	7.2	0.3	0.1	2.3	0.9	4.4
Carotid3	13 days	69	131	425	13.1	9.5	0	0	2.5	1.1	3.9
Carotid4	9 days	70	133	250	7.4	5.3	0.1	0	1.5	0.5	4.5
Carotid5	6 days	122	154	269	8.4	5.5	0.1	0	1.9	0.9	5.3
Carotid6	25 days	67	129	225	6	4.6	0.1	0	0.9	0.4	4.8
Carotid7	106 days	82	165	302	5.3	3.4	0.1	0	1.2	0.6	5.1
Carotid8	9 days	80	150	294	8.5	5.2	0.1	0	2.7	0.6	4.9
Limb1	3 days	69	125	501	10.5	7.5	0.1	0.1	2.2	0.8	4.0
Limb2	4 days	95	174	306	7.1	5.3	0.1	0	1.2	0.4	6.0
Limb3	>6 weeks	59	99	459	13.6	10.7	0	0	1.6	1.2	3.0
Limb4	6 days	70	83	163	8.3	6.1	0	0	1.5	0.6	2.7
Limb5	16 days	91	162	309	7.8	4.1	0.1	0.1	3.0	0.6	5.3
Limb6	15 days	63	179	313	9.2	6.3	0.5	0.1	1.0	1.3	5.8

Supplementary Table 4: MRM transitions for eoxPL analysed from the healthy cohort.

Name (ID)	Precursor m/z (Q1)	Product ion m/z (Q3)	RT
PE 16:0p_HETE	738.6	319.2	12.59
PE 16:0a_HETE	754.6	319.2	12.02
PE 16:0p_20:4;2O	754.6	335.2	10.95
PE 16:0p_HDoHE	762.6	343.2	12.02
PE 18:1p_12-HETE	764.6	179.1	12.90
PE 18:0p_20:5;O	764.6	317.2	14.22
PE 16:0p_22:5;O	764.6	345.2	12.50
PE 18:0p_12-HETE	766.6	179.1	14.10
PE 18:0p_15-HETE	766.6	219.1	13.63
PE 16:0e_22:5;O	766.6	345.2	12.92
PE 16:0p_22:4;O	766.6	347.2	13.28
PE 16:0a_20:4;2O	770.6	335.2	10.95
PE 16:0p_20:4;3O	770.6	351.2	9.40
PE 16:0p_Di-EHEDA	770.6	351.2	10.03
PE 18:2a_HETE	778.6	319.2	11.18
PE 16:0a_HDOHE	778.6	343.2	11.42
PE 18:1a_12-HETE	780.6	179.1	12.25
PE 18:0a_20:5;O	780.6	317.2	16.08
PE 18:1p_20:4;2O	780.6	335.2	10.93
PE 16:0a_22:5;O	780.6	345.2	11.98
PE 16:0p_22:5;2O	780.6	361.2	10.93
PE 18:0a_12-HETE	782.6	179.1	13.41
PC 16:0a_12-HETE	782.6	179.1	11.79
PE 18:0p_HDOHE	790.6	343.2	13.72
PE 18:1p_22:5;O	790.6	345.2	12.91
PE 18:0p_22:5;O	792.6	345.2	14.24
PE 18:1p_22:4;O	792.6	347.2	13.90
PE 20:0p_HETE	794.6	319.2	13.23
PE 18:0p_22:4;O	794.6	347.2	15.32
PE 18:1p_20:4;3O	796.6	351.2	9.80
PE 18:0p_20:4;3O	798.6	351.2	11.14
PE 18:1p_Di-EHEDA	796.6	351.2	10.53
PE 18:0p_Di-EHEDA	798.6	351.2	11.73
PE 18:1a_HDoHE	804.7	343.2	12.06
PC 18:2a_HETE	806.7	319.2	11.18
PC 16:0a_HDOHE	806.7	343.2	11.61
PE 18:0a_HDOHE	806.7	343.2	13.26
PE 18:1a_22:5;O	806.7	345.2	12.29
PE 20:1a_12-HETE	808.7	179.1	12.16
PC 18:1a_12-HETE	808.7	179.2	12.17
PC 16:0a_22:5;O	808.7	345.2	11.89
PE 18:0a_22:5;O	808.7	345.2	13.51
PE 18:1a_22:4;O	808.7	347.2	13.23
PC 18:0a_12-HETE	810.7	179.1	13.41
PE 18:0a_20:4;3O	814.7	351.2	10.35
PE 18:0a_Di-EHEDA	814.7	351.2	11.00
PE 18:0p_20:4;2O	782.6	335.2	13.28
PE 16:0a_22:4;O	782.6	347.2	12.80
DMPC (PC 14:0/14:0)	662.5	227.1	13.63
DMPE (PE 14:0/14:0)	634.5	227.1	13.98

Supplementary Table 5: MRM transitions for the oxPL analysed from the clinical cohort and clot samples

Analyte	Precursor m/z (Q1)	Product ion m/z (Q3)
DMPC (PC 14:0/14:0)	662.5	227.1
DMPE (PE 14:0/14:0)	634.5	227.1
PE 16:0p_5-HETE	738.6	115.1
PE 16:0p_12-HETE	738.6	179.1
PE 16:0p_15-HETE	738.6	219.1
PE 16:0p_11-HETE	738.6	167.1
PE 16:0p_8-HETE	738.6	155.1
PE 18:1p_5-HETE	764.6	115.1
PE 18:1p_12-HETE	764.6	179.1
PE 18:1p_15-HETE	764.6	219.1
PE 18:1p_11-HETE	764.6	167.1
PE 18:1p_8-HETE	764.6	155.1
PE 18:0p_5-HETE	766.6	115.1
PE 18:0p_12-HETE	766.6	179.1
PE 18:0p_15-HETE	766.6	219.1
PE 18:0p_11-HETE	766.6	167.1
PE 18:0p_8-HETE	766.6	155.1
PE 18:0a_5-HETE or PC 16:0a_5-HETE	782.6	115.1
PE 18:0a_12-HETE or PC 16:0a_12-HETE	782.6	179.1
PE 18:0a_15-HETE or PC 16:0a_15-HETE	782.6	219.1
PE 18:0a_11-HETE or PC 16:0a_11-HETE	782.6	167.1
PE 18:0a_8-HETE or PC 16:0a_8-HETE	782.6	155.1
PC 18:0a_5-HETE	810.7	115.1
PC 18:0a_12-HETE	810.7	179.1
PC 18:0a_15-HETE	810.7	219.1
PC 18:0a_11-HETE	810.7	167.1
PC 18:0a_8-HETE	810.7	155.1

Supplementary Table 6: MRM transitions for eicosanoids used for LC/MS/MS

Analyte	MRM transition	DP (V)	CE (V)	CXP (V)
12-HETE	319.2→179.1	−65	−18	−12
TxB2	369.1→169.1	−60	−22	−12
12-HETE-d8	327.2→184.1	−60	−20	−12
TxB2-d4	373.3→173.3	−55	−22	−10

Supplementary Table 7: Multiple reaction monitoring (MRM) transitions for chiral analysis of hydrolyzed HETE stereoisomers.

Analyte	MRM transition	DP (V)	CE (V)	CXP (V)
15-HETE	319.2→219.1	−70	−20	−13
12-HETE	319.2→179.1	−75	−22	−9
11-HETE	319.2→167.1	−75	−24	−1
8-HETE	319.2→155.1	−70	−22	−9
5-HETE	319.2→115.1	−70	−22	−7
12(S)-HETE-d8	327.2→184.1	−80	−22	−11

Supplementary Table 8: Demographics for individual patients for aspirin and P2Y12 inhibitors.

Patient Group	Age	Gender	Aspirin	P2Y12		Patient Group	Age	Gender	Aspirin	P2Y12
ACS	56-60	Male	Yes	Yes		HC	51-55	Male	No	No
ACS	66-70	Female	Yes	Yes		HC	51-55	Male	No	No
ACS	71-75	Male	Yes	Yes		HC	71-75	Female	No	No
ACS	56-60	Male	Yes	Yes		HC	81-85	Male	No	No
ACS	51-55	Male	Yes	Yes		HC	66-70	Male	No	No
ACS	51-55	Male	Yes	Yes		HC	46-50	Female	No	No
ACS	76-80	Male	Yes	Yes		HC	71-75	Male	No	No
ACS	66-70	Female	Yes	Yes		HC	61-65	Female	No	No
ACS	51-55	Male	Yes	Yes		HC	61-65	Male	No	No
ACS	51-55	Male	Yes	Yes		HC	51-55	Male	No	No
ACS	61-65	Male	Yes	Yes		HC	61-65	Female	No	No
ACS	71-75	Male	Yes	Yes		HC	76-80	Male	No	No
ACS	71-75	Female	Yes	Yes		HC	76-80	Female	No	No
ACS	61-65	Female	Yes	Yes		HC	41-45	Female	No	No
ACS	51-55	Male	Yes	Yes		HC	66-70	Male	No	No
ACS	46-50	Male	Yes	Yes		HC	66-70	Female	No	No
ACS	61-65	Male	Yes	Yes		HC	76-80	Male	No	No
ACS	71-75	Male	Yes	Yes		HC	61-65	Male	No	No
ACS	56-60	Male	Yes	Yes		HC	81-85	Male	No	No
ACS	76-80	Male	Yes	Yes		HC	66-70	Female	No	No
ACS	56-60	Female	Yes	Yes		HC	66-70	Female	No	No
ACS	76-80	Female	Yes	Yes		HC	56-60	Female	No	No
ACS	71-75	Female	Yes	Yes		HC	61-65	Male	No	No
ACS	76-80	Male	Yes	Yes		HC	51-55	Male	No	No

Patient Group	Age	Gender	Aspirin	P2Y12		Patient Group	Age	Gender	Aspirin	P2Y12
CAD	71-75	Male	No	Yes		RF	71-75	Female	Yes	No
CAD	66-70	Male	Yes	No		RF	51-55	Female	Yes	No
CAD	51-55	Female	Yes	Yes		RF	76-80	Female	Yes	No
CAD	66-70	Female	Yes	No		RF	61-65	Female	Yes	No
CAD	46-50	Male	Yes	No		RF	61-65	Male	No	No
CAD	36-40	Female	Yes	No		RF	46-50	Female	Yes	No
CAD	71-75	Male	Yes	No		RF	71-75	Female	Yes	No
CAD	61-65	Male	No	Yes		RF	56-60	Male	Yes	No
CAD	66-70	Male	No	Yes		RF	56-60	Male	Yes	Yes
CAD	71-75	Male	No	No		RF	51-55	Male	Yes	No
CAD	56-60	Male	Yes	No		RF	66-70	Male	Yes	Yes
CAD	71-75	Male	Yes	No		RF	66-70	Male	No	No
CAD	56-60	Male	Yes	No		RF	56-60	Female	Yes	No
CAD	61-65	Male	Yes	No		RF	51-55	Male	Yes	No
CAD	61-65	Male	Yes	No		RF	66-70	Male	Yes	No
CAD	71-75	Male	Yes	No		RF	51-55	Female	Yes	No
CAD	61-65	Male	Yes	No		RF	51-55	Male	Yes	No
CAD	66-70	Male	No	Yes		RF	66-70	Male	Yes	Yes
CAD	71-75	Male	Yes	Yes		RF	66-70	Male	No	No
						RF	56-60	Female	Yes	No
						RF	56-60	Female	Yes	No
						RF	61-65	Female	Yes	No
						RF	46-50	Male	Yes	No
